# Robust detection of point mutations involved in multidrug-resistant *Mycobacterium tuberculosis* in the presence of co-occurrent resistance markers

**DOI:** 10.1371/journal.pcbi.1008518

**Published:** 2020-12-21

**Authors:** Julian Libiseller-Egger, Jody Phelan, Susana Campino, Fady Mohareb, Taane G. Clark

**Affiliations:** 1 The Bioinformatics Group, School of Water, Energy and Environment, Cranfield University, Bedford, UK; 2 University of Natural Resources and Life Sciences, Vienna, Austria; 3 Faculty of Infectious and Tropical Diseases, London School of Hygiene and Tropical Medicine, London, UK; 4 Faculty of Epidemiology and Population Health, London School of Hygiene and Tropical Medicine, London, UK; University of Zurich, SWITZERLAND

## Abstract

Tuberculosis disease is a major global public health concern and the growing prevalence of drug-resistant *Mycobacterium tuberculosis* is making disease control more difficult. However, the increasing application of whole-genome sequencing as a diagnostic tool is leading to the profiling of drug resistance to inform clinical practice and treatment decision making. Computational approaches for identifying established and novel resistance-conferring mutations in genomic data include genome-wide association study (GWAS) methodologies, tests for convergent evolution and machine learning techniques. These methods may be confounded by extensive co-occurrent resistance, where statistical models for a drug include unrelated mutations known to be causing resistance to other drugs. Here, we introduce a novel ‘cannibalistic’ elimination algorithm (“Hungry, Hungry SNPos”) that attempts to remove these co-occurrent resistant variants. Using an *M. tuberculosis* genomic dataset for the virulent Beijing strain-type (n = 3,574) with phenotypic resistance data across five drugs (isoniazid, rifampicin, ethambutol, pyrazinamide, and streptomycin), we demonstrate that this new approach is considerably more robust than traditional methods and detects resistance-associated variants too rare to be likely picked up by correlation-based techniques like GWAS.

## Author summary

Tuberculosis is one of the deadliest infectious diseases, being responsible for more than one million deaths per year. The causing bacteria are becoming increasingly drug-resistant, which is hampering disease control. At the same time, an unprecedented amount of bacterial whole-genome sequencing is increasingly informing clinical practice. In order to detect the genetic alterations responsible for developing drug resistance and predict resistance status from genomic data, bio-statistical methods and machine learning models have been employed. However, due to strongly overlapping drug resistance phenotypes and genotypes in multidrug-resistant datasets, the results of these correlation-based approaches frequently also contain mutations related to resistance against other drugs. In the past, this issue has often been ignored or partially resolved by either restricting the input data or in post-analysis screening—with both strategies relying on prior information. Here we present a heuristic algorithm for finding resistance-associated variants and demonstrate that it is considerably more robust towards co-occurrent resistance compared to traditional techniques. The software is available at https://github.com/julibeg/HHS.

## Introduction

Tuberculosis disease (TB), caused by bacteria in the *Mycobacterium tuberculosis* (Mtb) complex, is a major global public health burden. In 2018, the WHO reported around 10 million cases globally and 1.3 million deaths from TB [1]. The Mtb genome is 4.4 Mb in size, features a high (65%) GC-content and contains ∼4,000 genes [2]. It is subject to low mutation and recombination rates with little to no horizontal gene transfer [3]. Of the seven main lineages comprising the Mtb complex, four predominantly infect humans and have spread globally (Lineage 1: Indo-Oceanic, Lineage 2: East Asian, Lineage 3: East-Africa-Indian and Lineage 4: Euro-American) [4]. They can vary in virulence, transmissibility, and drug resistance as well as geographic distribution and spread [3, 5, 6]. Lineage 2, especially Beijing strains, have shown to be particularly mobile with evidence of recent spread from Asia to Europe and Africa [7, 8].

Mtb drug resistance is making the control of TB difficult. It was estimated that 558,000 cases in 2017 were resistant to the first-line drug rifampicin (RIF; RR-TB). Among these, 82% had additional resistance to the first-line drug isoniazid (INH), leading to multidrug-resistant TB (MDR-TB). 8.5% of MDR-TB cases were further resistant to one fluoroquinolone and one injectable second-line drug, leading to extensively drug-resistant TB (XDR-TB) [1]. Drug resistance in Mtb is almost exclusively due to mutations [mostly single nucleotide polymorphisms (SNPs) but also insertions and deletions (indels)] in genes coding for drug-targets or -converting enzymes [4]. Additionally, changes in efflux pump regulation may have an impact on the emergence of resistance and putative compensatory mechanisms have been described to overcome the fitness impairment that arises during the accumulation of resistance-conferring mutations [9]. Resistance-associated point mutations have been found for all first-line drugs [RIF (e.g. in the *rpoB* gene), INH (*katG, inhA*), ethambutol (EMB; *embB*), streptomycin (SM; *rpsL, rrs*, and *gidB*), pyrazinamide (PZA; *pncA*)] as well as for fluoroquinolones (*gyrA* and *gyrB*) and other second-line drugs or injectables [ethionimide (*ethA*), bedaquiline (*rv0678*), amikacin (*rrs*), capreomycin (*rrs, tlyA*), kanamycin (*rrs, eis*)], but the understanding of resistance mechanisms is still incomplete [9].

Advances in whole-genome sequencing (WGS) are assisting the efforts to profile Mtb for drug resistance, lineage determination, virulence, and presence in a transmission cluster [10, 11], thereby informing clinical management and control policies. The use of WGS can reaffirm known resistance mutations and uncover new candidates through genome-wide association studies (GWAS) and convergent evolution analysis [9, 12, 13]. GWAS approaches assess a probabilistic association of a given allele with a phenotype, but need to account for the clonal nature of microbial genomes affecting population stratification, effective sample size, and linkage disequilibrium [9, 14]. Clonal populations, like those in Mtb, can be accounted for by sorting significant associations by lineage effects [6, 15], applying metric multidimensional scaling on inter-sample distances [4, 16], linear mixed models [9, 17], or employing compacted De Bruijn graphs [18].

The complementary convergent evolution approach aims at detecting the strong selection for drug resistance, which can be quantified by counting SNPs that have occurred independently on multiple occasions (i.e. homoplastic variants). The discrepancy in frequency of homoplastic events between resistant and susceptible branches of a phylogenetic tree can be used to estimate how strongly a mutation has been selected for. This approach inherently accounts for population structure and linkage disequilibrium in addition to being more robust towards small sample sizes [14].

Traditionally, estimating TB drug resistance has been based on known and biologically established mutations [3, 10]. However, as resistance phenotype prediction from genomic data is a binary classification problem with high-dimensional input—a standard task in statistical and machine learning (ML)—various such techniques have been applied to antibiotic resistance recently [19–26]. Employing ML in genomic phenotype prediction has two main advantages. First, several recent studies have shown that these techniques can at least compete with existing direct association methods based on mechanistic and evidential knowledge, which has been curated and scrutinised for decades [20–22, 24–26]. Second, examining important feature sets of the trained models might hint at yet unexplored variants leading to novel discoveries or reveal latent multidimensional interactions (e.g. compensatory mutations, epistasis) too subtle to be picked up by traditional techniques.

However, there are at least three challenges with ML applications for Mtb to date. First, co-occurrent resistance mutations are incorporated into the predictive models, potentially leading to overestimation of model performance which may not translate optimally into clinical practice [26]. Although the removal of unrelated genetic regions, adjustment for preceding resistance, or focusing on mono-resistance may remove bias, this leads to data size reduction and may be difficult to implement when prior knowledge about key mutations and loci is limited. Second, different models have been used for different drugs within the same study [23], rather than proposing a unified approach across all drugs [26]. Third, genomic data is often sparse, multicollinear and high-dimensional, where often a very small number of predictors explain most of the variance in the phenotype. Hence, preventing the models from overfitting by suitable regularisation strategies is paramount.

In light of the latest advances in bacterial GWAS as well as the plethora of ML algorithms available for binary classification, we sought to (i) compare the utility of some of these methods to re-discover point mutations that are already known to confer resistance, (ii) examine their robustness against co-resistant markers, and (iii) assess the accuracy of predicted resistance phenotypes. Further, we introduce a novel tool for finding resistance-conferring variants in strongly co-resistant datasets. In particular, we developed a procedure consisting of two stages. First, an initial batch of alleles is pre-selected by calculating a simple scoring heuristic to reduce the influence of population structure. The selected variants are then subjected to iterative ‘cannibalism’ until a final set of survivors remains. In reference to a popular table-top game, the method was named “Hungry, Hungry SNPos”. Here, we show its potential by applying it to a strongly MDR-TB dataset consisting of ∼41,000 missense SNPs in ∼3,600 strains of the Beijing sublineage of Mtb and compare the results with multiple GWAS implementations as well as the features most relevant for prediction in various ML models.

## Methods

### Data preparation

We used a subset of a global collection of ∼19,000 Mtb samples with whole-genome sequencing data from which ∼600,000 SNPs were derived [10] (see the ‘Additional file 2’ in ref. [10] for a list of accession codes and references). In brief, raw sequence data were mapped to the H37Rv reference genome with *bwa-mem*. From the alignments, variants were called using the *GATK* and *samtools* [27] software suites. Because we wished to focus on virulent strains and reduce the size of dataset (non-synonymous SNPs constituted less than half of all called variants), all Beijing strains (n = 3,574) and their polymorphic missense SNPs (41,319) were selected. Alleles were converted into binary genotypes, where ‘0’ represents the reference genotype and ‘1’ a missense-SNP at the respective genomic position. To check for biases in the missense SNPs versus the unfiltered Beijing dataset, the corresponding distributions of variants, allele counts and missing genotypes were plotted and principal component analysis (PCA) was performed on both matrices.

Although the dataset contained categorical resistance data for 15 antibiotics, most strains had only been phenotyped for the most common first-line drugs. Hence, this work only considered INH, RIF, EMB, PZA, and SM. Strains with known resistance status for at least one of these five drugs were selected for analysis. Each of the resulting six datasets (one per drug and one including all drugs) was filtered to remove SNPs and samples with more than 10% uncalled genotypes as well as mono-allelic sites. For all analyses intolerable to missing data the corresponding genotypes were set to the respective allele frequency.

Strain metadata including resistance status to the five drugs are available in S6 Data. The filtered genotype matrix can be found in S7 Data.

### Phylogenomics

A phylogenetic tree was obtained from the ∼41,000 missense SNPs using *fasttree* (v2.1.11) [28] with a Generalised Time Reversible (GTR) substitution model and branch length rescaling to optimise the Gamma20 likelihood. The multi-FASTA alignment file required for running *fasttree* was generated from the called variants using *vcf2phylip* (v2.0) [29]. A proto-Beijing strain (ERR751993) belonging to lineage 2.1 was presented as the outgroup. The resulting tree topology was then evaluated under various model parameters with *RAxML-NG* (v0.9.0) [30] (the evaluation step re-optimises the branch lengths under a given model). For all subsequent analyses requiring phylogenetic input, the tree with the lowest AIC metric [31] was used. It was created using GTR with the Gamma model for rate heterogeneity and estimated base frequencies. The tree was visualised with *ggtree* (v1.16.3) [32]. The command line arguments used for *fasttree* and *RAxML-NG* are listed in S1 Methods.

### Genome-wide association studies

GWAS was performed with multiple ways of correcting for population structure, including multidimensional scaling (MDS) as implemented in *SEER* [16], linear mixed models as in *FaST-LMM* [17] and lineage effects as in *bugwas* [15]). These analyses were run via the *pyseer* package [33]. *SEER* was run once with four and once with ten dimensions to be included in MDS. The pairwise distance as well as similarity matrices were generated from the phylogenetic tree by the respective scripts coming with *pyseer*. Homoplastic variants were tested for convergent evolution with *treeWAS* [13]. We provided the phylogenetic tree employed in the analysis, whereas the ancestral reconstruction was performed by *treeWAS* internally. The command line arguments passed to *SEER* can be found in S1 Methods.

### Statistical and machine learning

Most ML methods employed were available via Python’s *sklearn* package (v0.21.2) [34]. These included regularised linear regression (L1 penalised; i.e. Lasso [35]), logistic regression (L1 and L2 penalised), support vector machines [SVMs; using linear and radial basis function (RBF) kernels] [36], decision trees [37], random forests [38], and gradient boosted decision trees [39]. Neural networks (NNs) were implemented with *Keras* (v2.2.4) [40] upon *TensorFlow* (v1.13.1) [41]. Model parameters are described in more detail in S2 Methods and the NN architecture is illustrated in S1 Fig.

All algorithms were applied on the five single-drug datasets separately. Additionally, an extra NN was trained for multi-label classification on the dataset with all drugs. Thus, it would predict resistance against one or more of the five drugs simultaneously. Each dataset was split into training and testing batches stratified for phenotype at a ratio of 0.7 to 0.3. The models were then trained on the training data and benchmarked on the testing data. During training of the NNs, 20% of the training data were used for validation. For selecting the optimal shrinkage parameters for Lasso and logistic regressions, *sklearn*’s respective cross-validation functions were utilised. Decision trees were left at default parameters (representing minimum restriction) or constrained to a maximum depth of five. Hyperparameters for random forests and gradient boosted trees were selected with *sklearn’s* cross-validated grid-search method according to the F_1_-score [42] as scoring metric. Class weights were balanced wherever possible.

For Lasso and linear SVM the absolute magnitude of model coefficients was interpreted as feature importance. For logistic regression, a likelihood-ratio test was implemented [43, 44]. The test calculates two *p*-values for a given feature according to two different approaches: (i) the corresponding regression coefficient is set to zero and the resulting difference in log-likelihood of the prediction of the training set is subjected to a *χ*^2^ test; (ii) the model is fit on the respective feature alone and the difference of the prediction log-likelihood to the raw class probabilities is subjected to a *χ*^2^ test. *p*-values were calculated for the 1000 features with the largest absolute regression coefficients per model.

For all other *sklearn* models (except for SVM with RBF kernel) feature importance values were determined by the respective implementation internally. From NNs or SVMs with non-linear kernels feature importance cannot be quantified directly, but must be estimated by monitoring prediction accuracy while permuting the input data [45]. However, this requires predicting a considerable number of samples at least once per feature and thus is computationally infeasible given the size of the datasets used in this work.

### Hungry, Hungry SNPos algorithm (HHS)

To minimise the confounding effects of co-occurrent resistance markers, we formulated an iterative, ‘cannibalistic’ elimination algorithm. In its first stage an initial score for each SNP *i* is calculated according to

$$\begin{array}{c} {\text{score}}_{i}=(p^{1}g_{i}^{1}-p^{0}g_{i}^{1}\cdot w_{p^{0}g^{1}})\cdot d_{p^{1}g^{1},i}\\ \text{with}\;p^{1}g_{i}^{1}=\sum_{j=1}^{n}p_{j}\cdot g_{i,j}\;\text{and}\;p^{0}g_{i}^{1}=\sum_{j=1}^{n}\left(1-p_{j}\right)\cdot g_{i,j} \end{array}$$ (1)

with the (binary) phenotype *p*, the (binary) genotype *g*, the number of samples *n*, *d*_*p*^1^*g*^1^, *i*_ as a distance measure for how closely related all strains with *p*_*j*_ = 1 and *g*_*i*,*j*_ = 1 are (e.g. the average pairwise Hamming distance or an average distance extracted from a phylogeny), and *w*_*p*^0^*g*^1^_ as a weight factor that can be set to values > 1 to exaggerate the effect of strains that are not resistant but still have the genotype.

The rationale behind this formulation as opposed to the scores calculated by *treeWAS* is that *p*^1^ *g*^0^ and *p*^0^ *g*^0^ add little relevant information for assessing a particular variant as resistance in strains with *p*^1^ *g*^0^ could be caused by other SNPs (which we did not want to penalise) and because *p*^0^ *g*^0^’s relation to a SNP’s association with resistance is typically weak. *p*^1^ *g*^1^ favours high-frequency alleles in imbalanced datasets with a large proportion of resistant strains, but this can be counteracted by a sufficiently high *w*_*p*^0^*g*^1^_. In the actual implementation, additional weights can be applied to normalise for the overall allele frequency at the respective site and class imbalance in the phenotype. The weight of the distance *d*_*p*^1^*g*^1^, *i*_ is tunable as well. See S3 Methods for a more detailed description of the different parameters.

After this first step all SNPs with positive scores are subjected to iterative elimination as outlined in S3 Methods. In every iteration, the procedure reduces the score of every SNP *i* by an amount that is proportional to (i) the overlap of *i* with other SNPs across all resistant strains and (ii) the magnitude of these other SNPs’ scores. After each iteration, all scores are rescaled so that their overall sum stays the same. Thus, some scores will grow and others will shrink. As soon as a score drops below zero, it is no longer considered. After a certain number of iterations, a stable set of SNPs remains.

The results reported below were generated with a Python prototype relying on *NumPy* [46, 47], *SciPy* [48], *pandas* [49], and *Numba* [50]. First, the pairwise Hamming distances between all strains in the dataset were calculated. Then, HHS was run on the five single-drug datasets in various different configurations (for details see S3 Methods) for 20,000 iterations each. This was enough to let the algorithm converge (i.e. the scores would no longer change). A Rust [51] implementation optimised for memory efficiency (with a footprint of $∼1/4\times n_{\text{samples}}\times n_{\text{SNPs}}+2\times n_{\text{samples}}^{2}$ bytes) is available at https://github.com/julibeg/HHS. A run of 20,000 iterations (e.g. to test a specific set of parameters) with the Beijing dataset took about 20 seconds (or 10, if the pairwise distances were already known) on a standard laptop, whereas hours of high-performance computing were required for some of the other methods tested herein.

## Results and discussion

### Mtb Beijing strain data and phylogeny

The Beijing strains (n = 3,574) were from 43 countries, with nearly half (n = 1771; 49.6%) from Thailand, South Africa and Russia (S2 Fig). To check for systematic biases within the set of missense SNPs, the distributions of variants, non-calls and allele-counts were compared with the original dataset holding all SNPs and no striking differences were found (S3 and S4 Figs).

**Fig 1:**
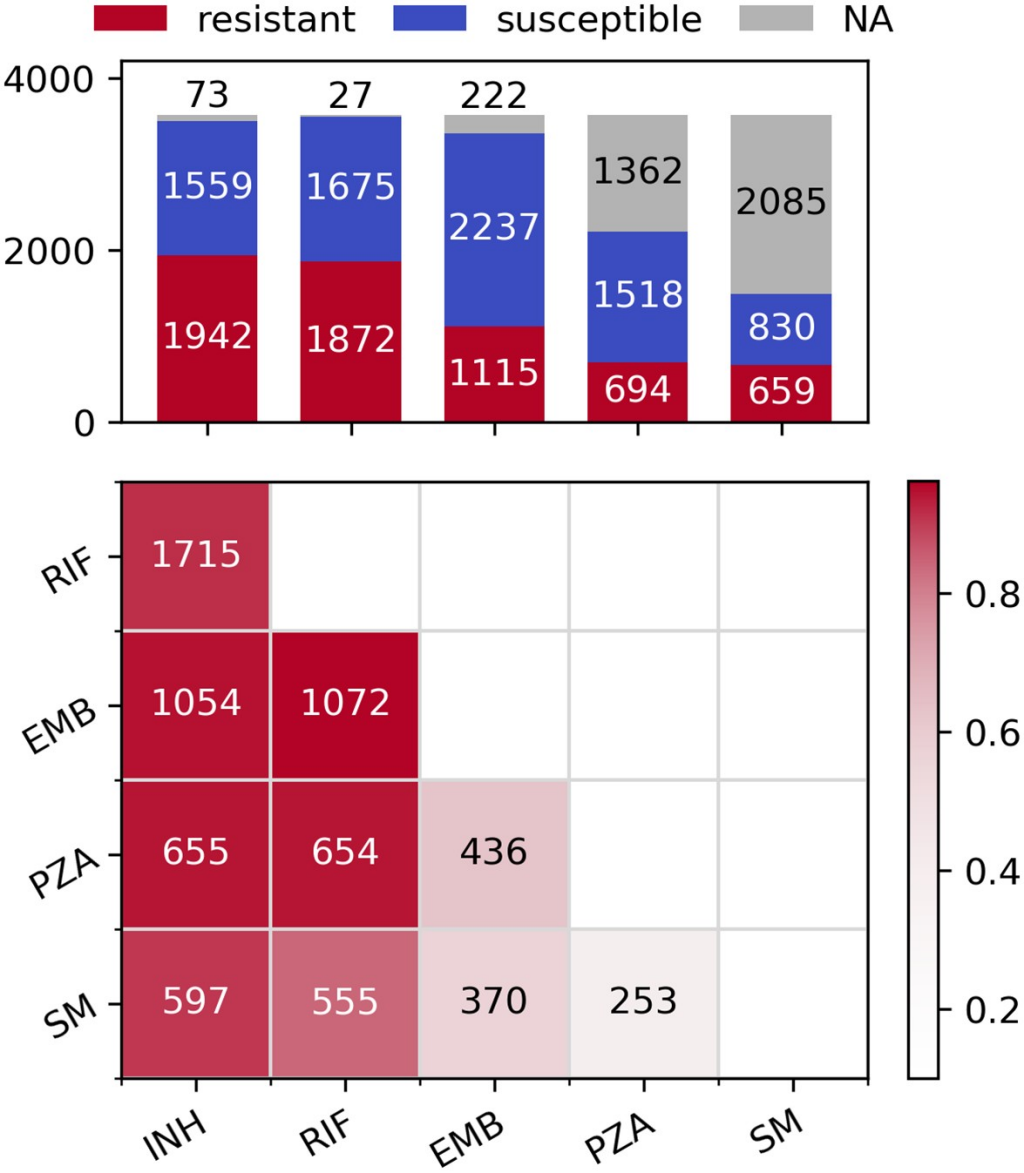
Distribution of resistance phenotypes. Top: Bar plot of resistant, susceptible and missing phenotypes per drug. Bottom: Pairwise co-occurring resistance for the five drugs in the dataset. Colours are relative to the drug with fewer resistant strains (e.g. 597/659 = 0.906 for SM and INH, 1072/1115 = 0.961 for EMB and RIF etc.). The upper triangle of the symmetric matrix has been removed for clarity.

For each of the five drugs, sample sizes varied from 1489 (SM) to 3547 (RIF), leading to between 24,946 and 41,040 SNPs being analysed, respectively (Table 1). The differences are driven by the number of susceptibility tests performed for each drug across the 3,574 samples (Fig 1, top). There is a high level of co-occurring resistance, especially between partner drugs such as INH and RIF, leading to a high proportion of the samples being MDR-TB (Fig 1, bottom). This makes detecting resistance-conferring SNPs difficult as mutations strongly associated with resistance to one drug (e.g. *katG* S315N/T for INH) also give large association signals when analysing other drugs.

**Table 1:** Specifics of the datasets used.

Dataset	N	% resistant	Training N	Testing N	SNPs
All drugs	3574	60.9	2501 (501)	1073	41319
INH	3501	55.5	2450 (490)	1051	41040
RIF	3547	52.8	2482 (497)	1065	41120
EMB	3352	33.3	2346 (470)	1006	40252
PZA	2212	31.4	1548 (310)	664	22403
SM	1489	44.3	1042 (209)	447	24946

The size of the respective training subset used for validation in neural net hyperparameter search is given in brackets. N, number of samples.

**Fig 2:**
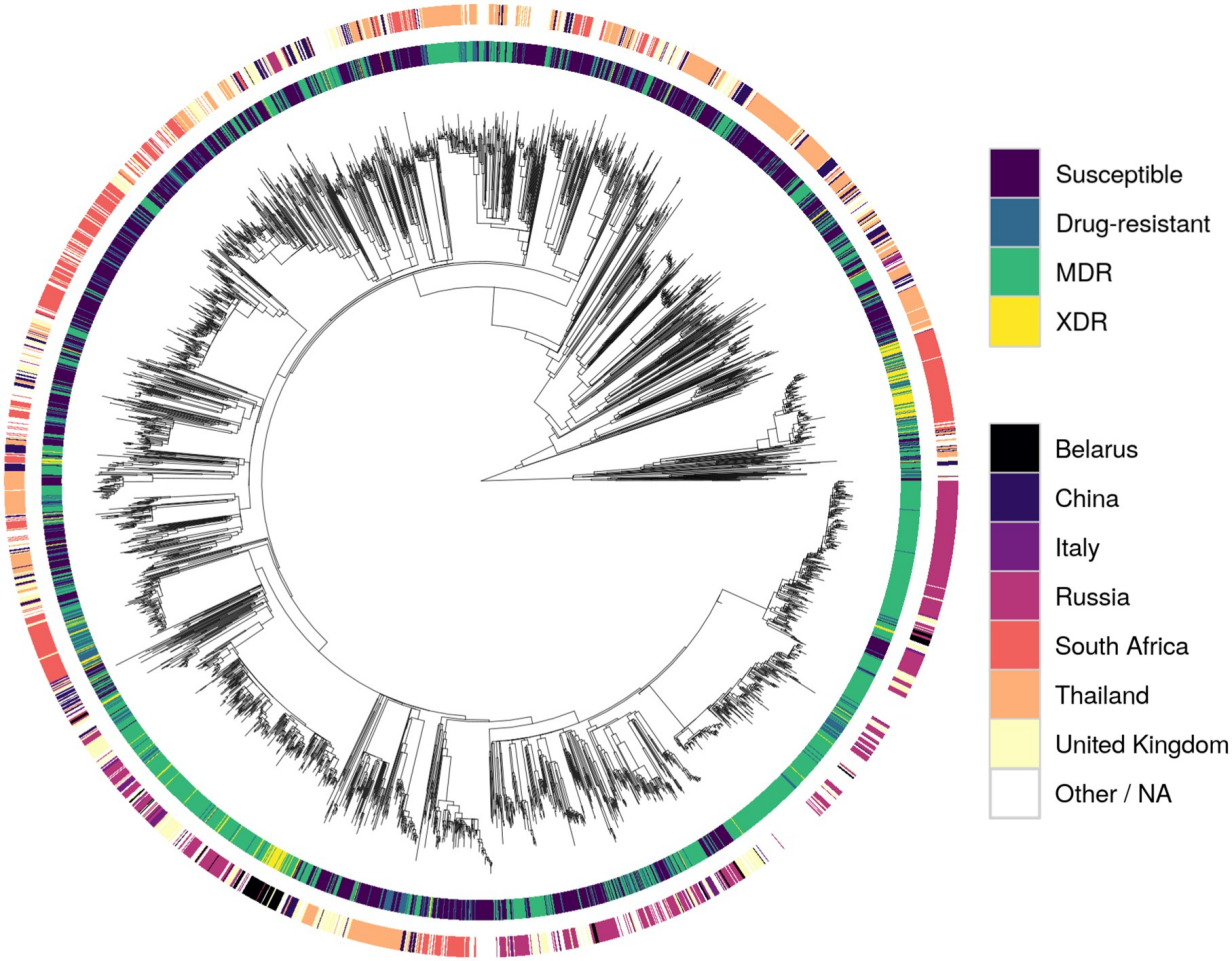
Phylogeny of the dataset. Tracks are coloured by resistance status (inner track) and source country of Mtb (outer track). The root node was omitted for clarity.

A phylogenetic tree inferred from the 41,319 missense SNPs revealed that the XDR strains clustered relatively well, with evidence of potential transmission clusters for single countries (South Africa and Belarus; Fig 2). Otherwise, samples with the same resistance status or that originated from the same country spread throughout the tree. This was also found in a PCA (S5 Fig), with the majority of clusters being heterogeneous.

### Resistance mutations found using GWAS and phylogeny testing

The four SNPs most significantly associated with each drug as determined by GWAS with *FaST-LMM* are presented in Table 2. Similarly, the significant SNPs for *SEER* are listed in S1 and S2 Tables. The results produced by *FaST-LMM* featured the least number of co-resistant false positives, which might be attributed to it being less prone to inflating *p*-values as opposed to *SEER* [52]. The *SEER* results showed only minimal difference between projecting the phylogenetic distances onto four or ten dimensions.

**Table 2:** Most significant hits according to GWAS.

Drug	Gene	Position	Codon	Ref. AA	*p*^1^ *g*^1^	*p*^0^ *g*^1^	*p*-value	Resistance
INH	*katG*	2155168	315	S	1676	12	8.19E-217	INH
	*rpsL*	781687	43	K	1118	79	6.58E-54	SM
	*rpoB*	761155	450	S	1289	54	1.78E-38	RIF
	*embB*	4247429	306	M	583	24	4.88E-18	EMB
RIF	*rpoB*	761155	450	S	1367	9	4.46E-153	RIF
	*katG*	2155168	315	S	1548	169	2.52E-74	INH
	*rpoB*	761139	445	H	135	4	3.32E-34	RIF
	*rpsL*	781687	43	K	1044	162	2.34E-32	SM
EMB	*katG*	2155168	315	S	1004	587	1.89E-75	INH
	*embB*	4247429	306	M	455	120	2.40E-69	EMB
	*rpoB*	761155	450	S	854	433	1.99E-65	RIF
	*rpsL*	781687	43	K	729	381	1.16E-42	SM
PZA	*rpoB*	761155	450	S	549	452	5.43E-28	RIF
	*katG*	2155168	315	S	623	558	5.31E-22	INH
	*embB*	4247429	306	M	292	160	1.61E-17	EMB
	*rpsL*	781687	43	K	446	378	1.71E-10	SM
SM	*rpsL*	781687	43	K	469	11	5.91E-121	SM
	*katG*	2155168	315	S	542	80	2.44E-35	INH
	*rpsL*	781822	88	K	60	7	8.61E-24	SM
	*rpoB*	761155	450	S	411	74	3.82E-14	RIF

Top four SNPs per drug as reported by *pyseer’s* implementation of *FaST-LMM*. SNPs from genes known to be involved in resistance to the respective drug are shaded red. SNPs from genes known to be involved in resistance to other drugs are shaded blue. Ref. AA, reference amino acid.

Although at least one known resistance-conferring SNP ended up in the top four for every drug except PZA, the results were strongly confounded by false positives due to co-occurring resistance. For PZA, the most significant SNP in *pncA* (the gene coding for the enzyme activating PZA) was ranked 7th, 36th, and 75th for *Fast-LMM* and the two *SEER* settings, respectively. The challenge of rediscovering SNPs that are actually related to PZA resistance can be at least partially attributed to *pncA* featuring a larger number of rather uncommon resistant alleles as opposed to genes with very few but prominent variants like S315T/N in *katG* conferring resistance to INH [6]. More results produced by the two GWAS methods can be found in S1 Data.

Sorting SNPs not only by significance but also lineage as done by *bugwas* [15] leads to a higher portion of true positives reported for some lineages (S2 Data). Albeit useful, this does not fundamentally solve the issue of co-occurring resistance as other lineages are even more strongly confounded in return.

The significant SNPs reported by *treeWAS* draw a very similar picture (S3 Table). The fact that this method, which follows a very different approach for handling population structure and significance testing, also suffers from false positives due to co-occurring resistance, underlines the general nature of this frequently neglected problem.

### Application of machine learning for predicting drug resistance

**Fig 3:**
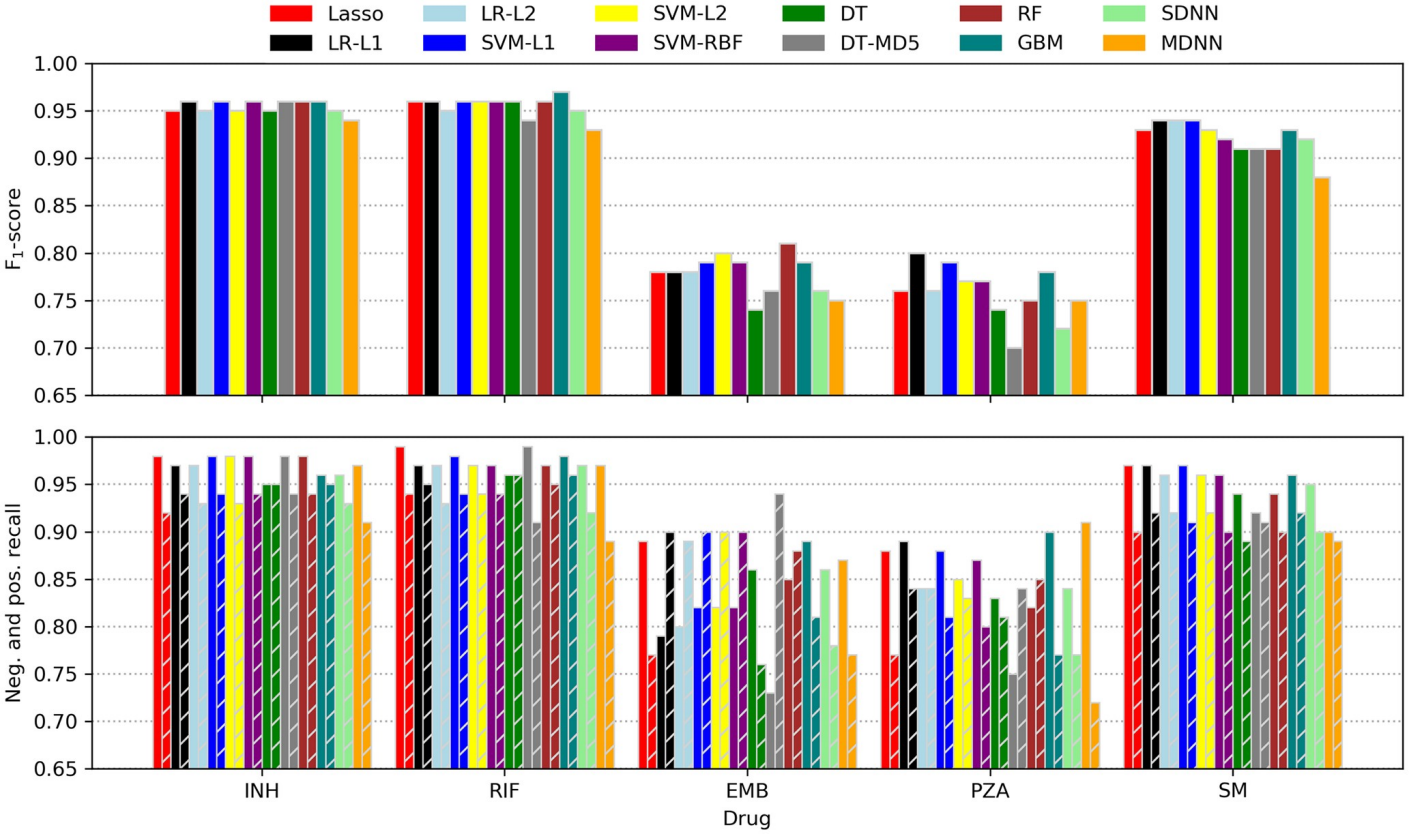
Machine learning prediction performance. F_1_-scores (top) as well as negative and positive recalls (bottom; left and right bars of corresponding colour) of all classifiers tested. LR, logistic regression; L1, L1 penalty; L2, L2 penalty; SVM, support vector machine; RBF, radial basis function kernel; DT, decision trees; MD5, maximum depth of five; RF, random forest; GBM, gradient-boosted machines; SDNN, single-drug neural net; MDNN, multidrug neural net.

Although the F_1_-score was used for ranking models while searching hyperparameter space, the more intuitive recall metric was additionally employed for assessing classifier performance. Calculated for the positive (resistance) and negative (susceptible) case separately, recall is equivalent to the sensitivity or true-positive rate (positive recall) and specificity or true-negative rate (negative recall), respectively. Performance metrics of all models tested are depicted in Fig 3 and listed in Table 3.

**Table 3:** ML prediction performance.

Method	INH			RIF			EMB			PZA			SM			Package	Studies
	F_1_	R+	R−	F_1_	R+	R−	F_1_	R+	R−	F_1_	R+	R−	F_1_	R+	R−		
Lasso	.95	.92	.98	.96	.94	.99	.78	.77	.89	.76	.77	.88	.93	.90	.97	*sklearn*	–
LR-L1	.96	.94	.97	.96	.95	.97	.78	.90	.79	.80	.84	.89	.94	.92	.97	*sklearn*	[21–23, 26]
LR-L2	.95	.93	.97	.95	.93	.97	.78	.89	.80	.76	.84	.84	.94	.92	.96	*sklearn*	[19, 22–24]
SVM-L1	.96	.94	.98	.96	.94	.98	.79	.90	.82	.79	.81	.88	.94	.91	.97	*sklearn*	–
SVM-L2	.95	.93	.98	.96	.94	.97	.80	.90	.82	.77	.83	.85	.93	.92	.96	*sklearn*	[22, 23, 25]
SVM-RBF	.96	.94	.98	.96	.94	.97	.79	.90	.82	.77	.80	.87	.92	.90	.96	*sklearn*	[20, 22, 23]
DT	.95	.95	.95	.96	.96	.96	.74	.76	.86	.74	.81	.83	.91	.89	.94	*sklearn*	[26]
DT-MD5	.96	.94	.98	.94	.91	.99	.76	.94	.73	.70	.84	.75	.91	.91	.92	*sklearn*	[26]
RF	.96	.94	.98	.96	.95	.97	.81	.88	.85	.75	.85	.82	.91	.90	.94	*sklearn*	[19, 20, 23–25]
GBM	.96	.95	.96	.97	.96	.98	.79	.81	.89	.78	.77	.90	.93	.92	.96	*sklearn*	[19, 23, 26]
SDNN	.95	.93	.96	.95	.92	.97	.76	.78	.86	.72	.77	.84	.92	.90	.95	*Keras*	[19, 24]
MDNN	.94	.91	.97	.93	.89	.97	.75	.77	.87	.75	.72	.91	.88	.89	.90	*Keras*	[24]

Performance metrics of the ML models tested in addition to the underlying Python packages and recent studies employing similar techniques for antibiotic resistance prediction. F_1_, F_1_-score; R+, positive recall; R−, negative recall; other abbreviations are explained in Fig 3.

For INH, RIF, and SM most models performed similarly well with consistently higher recalls for the ‘susceptible’ class. This observation is not surprising as the resistance of some strains might have been caused by genomic features other than missense-SNPs like indels or SNPs in non-coding regions. Moreover, in some cases, resistance might have been conferred by cooperative networks of SNPs that all need to be present concomitantly in order to take effect. As such networks are likely to be ‘fluctuant’ (i.e. several similar patterns might yield comparable effects) as well as harder to obtain evolutionally (it is more likely to acquire a single or a few strongly selected mutations than a larger number of weakly selected, cooperative ones), they are underrepresented in the dataset and thus not learned effectively. Nonetheless, the recalls are comparable to what was reported in other recent resistance-prediction studies (e.g. [23]) and, for INH and RIF, also to a state-of-the art direct association model [10]. It should be kept in mind, though, that the different studies used different data and hence comparisons should be interpreted carefully.

Surprisingly, for SM all models outperformed the direct association approach [10], even though the rRNA gene *rrs* was completely absent from the missense-only dataset. However, for EMB and PZA both [23] and [10] outperformed the models tested here with the difference in performance being smaller for PZA. The performance gap between INH, RIF and SM on one hand and EMB and PZA on the other may be partially explained by the latter two datasets being the most imbalanced ones. Additionally, two of the ten most important SNPs for predicting EMB resistance reported by [23] lie outside the coding region of *embA* and are thus absent from the missense SNPs used here.

Although trained and tested on the same data, there is high variance among the performance metrics of the individual models for the harder-to-predict drugs EMB and PZA. This result could be attributed to stratification within the training/testing splits favouring some models over others. Repeating the fitting and testing process multiple times as done in some other studies [22, 23, 53] might smooth out the variation but was infeasible given the computational resources available for this work.

Remarkably, NNs did not outperform the simpler learning algorithms and the multidrug version did consistently worse than its single-drug counterparts. It should be noted, though, that the space of possible network architectures, regularisation methods, loss functions and optimisers with accompanying hyperparameters is tremendously vast and a proper hyperparameter search for deep NNs was not in the scope of this work. Nonetheless, despite exploring more complex NN architectures, Chen *et al*. also found that L2 regularised logistic regression performed on a par with their best neural nets [24]. In combination with the results reported here, this might indicate that—given the rather direct relationship between genotype and phenotype—the higher complexity of NNs is not justified. Overall, the results do not show a clear trend towards a single method or family of algorithms and most models performed comparably well.

**Fig 4:**
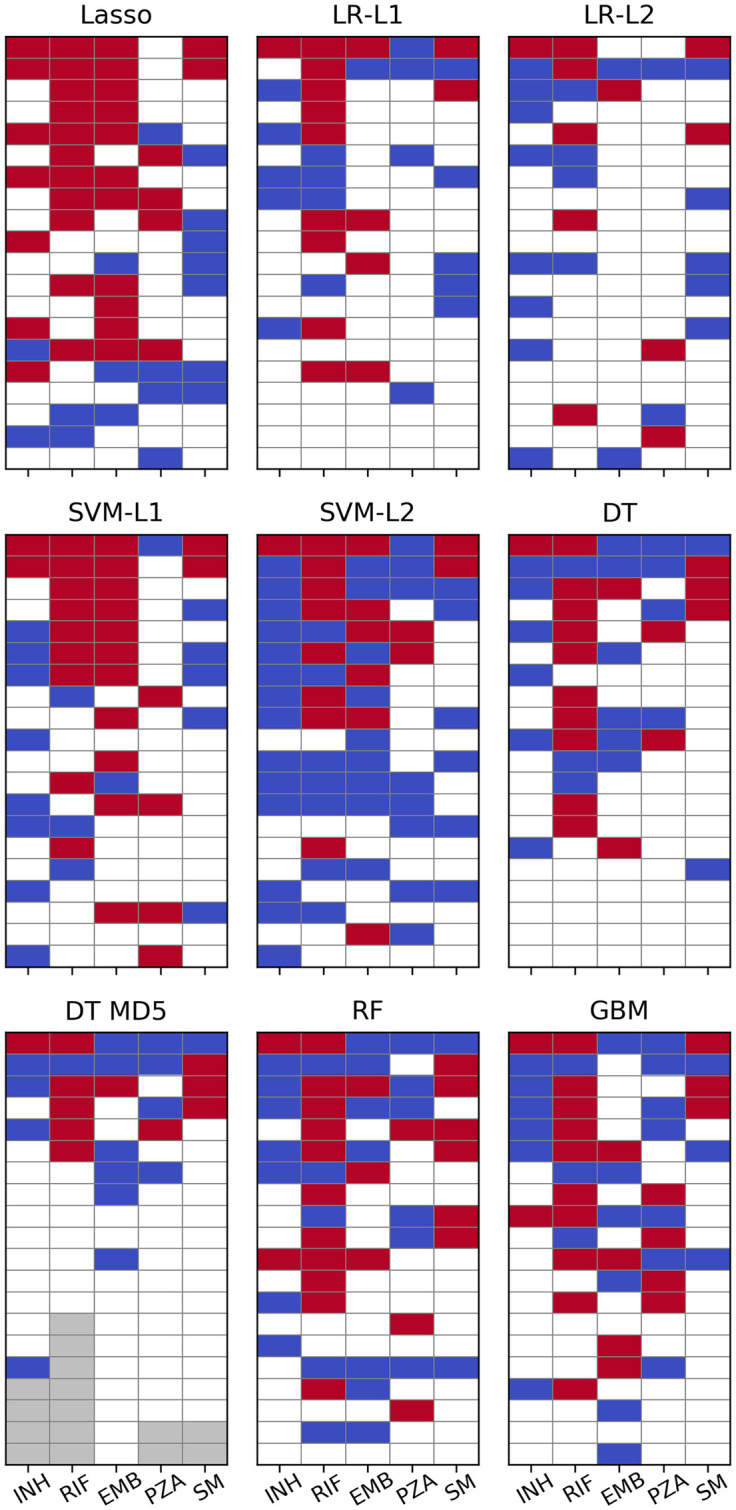
Machine learning feature importance. Top 20 SNPs per ML algorithm as sorted by the respective measure of feature importance. Cells are coloured red for SNPs in genes known to contribute to resistance to the respective drug and blue for SNPs involved in resistance to other drugs. Grey cells denote a feature importance of zero or a *p*-value of 1 (i.e. there are fewer than 20 SNPs with non-zero feature importance). *p*-values for LR have been calculated with approach 1.

The 20 most important SNPs of all classifiers allowing for extraction of feature importances are illustrated in Fig 4. Linear SVM and especially Lasso appeared to be less sensitive to co-occurring resistance, whereas all other models seemed to be affected equally with true and false positives occurring at roughly the same rate. Lasso’s greater robustness might be attributed to it being a regression rather than a classification method. Therefore, its output is not bounded between 0 and 1 and the model tries to keep the prediction for resistant strains as close to 1 as possible since overestimating the resistance status would increase the error just as much as underestimating it would. Conversely, all the other methods are implemented as proper classifiers only capable of producing class probabilities between 0 and 1. Thus, taking prediction of INH resistance as an example, giving large weight coefficients to S315 in *katG* as well as S450 in *rpoB*, which are both present in ∼1,200 strains, would let Lasso predict a number close to 2 for most samples, whereas logistic regression due to its sigmoid nature would only be even more sure that the sample is 1, i.e. resistant.

However, regression coefficients for strongly correlated genotypes (e.g. compensatory or cooperative mutations) are likely to be underdetermined and hence unstable in Lasso. This would make Ridge regression [ordinary least squares (OLS) with L2 penalty] [54] or Elastic Nets (OLS with linear combination of L1 and L2 penalties) [55] better choices for exploring feature importance in such cases as the quadratic penalty forces coefficients of collinear predictors to take similar values. These methods have not been tested systematically in this work, though.

For PZA not a single algorithm ascribed highest relevance to a SNP in *pncA* and in L1 regularised logistic regression the most important SNP from that gene appeared only at rank 23. It should be noted, however, that the plots for logistic regression in Fig 4 are based on the *p*-values calculated with approach 1 (see Methods) which should be viewed only as an easily available heuristic for feature importance. When sorted by absolute magnitude of regression coefficients, six *pncA* SNPs were featured in the top 20 (see S6 Fig and S4 Data).

The white tiles in Fig 4 represent “unknown” SNPs that have not been linked to resistance so far (we define “unknown” as not being present in the TBProfiler [10] database). They are listed in more detail in S4 Table and some show very favourable *p*^1^ *g*^1^ / *p*^0^ *g*^1^ ratios. However, upon closer inspection of their distribution across the phylogeny, for most variants the association appears to be due to population stratification. This is also evident from their relatively small average pairwise distance values. A SNP in a hypothetical protein (genome position 2238734), for instance, occurred in 78 samples resistant against RIF and only in seven that were susceptible. Yet, all strains carrying the mutation are located on the same clade in the phylogenetic tree (S7 Fig). Nonetheless, a few promising candidates were found (Table 4). P93 in *idsB*, for example, appeared in 12 INH-resistant and two susceptible strains and has emerged multiple times independently (S8 Fig). Moreover, nine of the 12 resistant samples also carried a resistance-conferring SNP in *katG*. Thus, one could argue that *idsB* P93 might have a compensatory effect reducing the fitness penalty incurred by the *katG* mutations.

**Table 4:** Potential novel variants related to resistance extracted from the ML feature importances.

Drug	Gene	Locus Tag	Position	Ref. AA	Codon	*p*^1^ *g*^1^	*p*^0^ *g*^1^	Distance	Method	Rank	Comment
INH	*idsB*	Rv3383c	3798212	P	93	12	2	0.688	SVM-L1	19	possibly compensatory for *katG*
RIF	.	Rv2348c	2626678	I	101	10	4	1.484	SVM-L1	18	strong homoplasy, but many susceptible strains and overlap with *rpoB* in resistant strains
	.	Rv3467	3884871	P	303	30	15	1.172	SVM-L2	19	
PZA	*rpoB*	Rv0667	761314	F	503	8	1	0.522	Lasso	16	probably false positive and actually related to RIF overlap with *pncA*
	.	Rv2670c	2986827	A	5	76	23	0.479	GBM	17	

The most promising SNPs with high *p*^1^ *g*^1^ / *p*^0^ *g*^1^ ratios and large distance values (indicating strong homoplasy) were selected from S4 Table Ref. AA, reference amino acid.

Further, eight samples resistant against PZA featured a mutation in *rpoB* F503. However, these were all resistant against RIF as well, which—since *rpoB* is the main target of RIF—might indicate a potential false positive. Nonetheless, *rpoB* F503 could still represent a novel compensatory variant related to RIF resistance as all strains carrying it also feature *rpoB* S450.

SNPs at the positions 2626678 and 3884871 (both in hypothetical proteins) showed strong homoplasy, but only two thirds of the strains they appeared in were resistant against RIF. Moreover, all 10 resistant samples with a SNP at 2626678 and 29 out of 30 with a SNP at 3884871 also carried at least one SNP in *rpoB*. Thus, although perhaps not causing resistance, these variants might provide some other fitness advantage, which has been strongly selected for recently, explaining their frequent independent emergence.

Similarly, position 2986827 (in a hypothetical protein) has also mutated independently multiple times and occurred in 76 strains that were resistant against PZA (vs. 23 susceptible ones). Sixty-one of the resistant samples carried *pncA* SNPs as well, possibly hinting at a compensatory or cooperative effect. However, the majority of strains featuring a SNP at that position lie within a single clade (S8 Fig). Hence, further research with complementary methods is required to confirm these associations.

### Application of Hungry, Hungry SNPos

The last SNPs remaining after 20,000 iterations for every drug as determined by HHS are listed in Table 5. As opposed to most of the other methods, the algorithm re-discovered predominantly true positives. The results in Table 5 were obtained with *w*_*p*^0^*g*^1^_ = 2, a filter ignoring all SNPs with *p*^1^ *g*^1^ < 5, normalised *p*^1^ *g*^1^ and *p*^0^ *g*^1^ values, and a distance weight of 1. This set of parameters turned out to be most reliable across the five drugs tested. Additional results are available in S5 Data.

**Table 5:** ’Last SNPos standing’ after 20,000 iterations of HHS.

Drug	Gene	Position	Codon	Ref. AA	*p*^1^ *g*^1^	*p*^0^ *g*^1^	Counts	Distance	*Score*_0_	*Score*_20000_	Resistance
INH	*katG*	2155541	191	W	10	0	1577.9	1.040	1640.4	237633.6	INH
	*katG*	2155169	315	S	8	0	1577.9	0.850	1337.1	159553.4	INH
	*katG*	2155168	315	S	1676	12	1538.7	0.945	1453.4	128838.5	INH
RIF	*rpoB*	761101	432	Q	10	0	1680.2	1.174	1973.1	135374.4	RIF
	*rpoB*	761128	441	S	13	0	1680.2	1.032	1733.6	107171.9	RIF
	*rpoB*	760314	170	V	24	0	1680.2	0.922	1549.1	93596.2	RIF
	*rpoB*	761139	445	H	135	4	1523.8	1.073	1634.5	74374.8	RIF
	*rpoB*	761155	450	S	1367	9	1644.6	0.844	1387.9	35940.8	RIF
	*rpoB*	761110	435	D	131	7	1404.5	1.328	1865.4	30883.7	RIF
EMB	*embB*	4247495	328	D	7	1	1890.4	1.446	2733.1	57535.3	EMB
	*gyrB*	6742	501	E	8	1	1960.3	1.217	2385.3	44699.3	FQ
	*rpoC*	765846	826	N	5	1	1680.8	0.884	1486.6	31274.1	RIF
	*embB*	4247729	406	G	13	3	1576.0	0.785	1236.5	23067.0	EMB
	*rpoB*	761097	431	S	5	0	2519.3	0.784	1975.7	22631.1	RIF
	*embB*	4247469	319	Y	11	3	1441.3	0.852	1228.0	16557.9	EMB
	*pncA*	2288703	180	V	7	1	1890.4	0.490	926.2	16378.0	PZA
	*embB*	4248003	497	Q	82	25	1343.9	0.956	1285.2	13938.8	EMB
	*embB*	4247429	306	M	455	120	1469.4	0.781	1147.5	11245.9	EMB
PZA	*pncA*	2289096	49	D	6	0	1762.6	0.967	1704.0	31191.7	PZA
	*hadA*	732110	61	C	5	0	1762.6	1.112	1960.5	27771.6	.
	*pncA*	2289043	67	S	7	1	1340.8	0.975	1307.3	15491.8	PZA
	*pncA*	2288839	135	T	9	0	1762.6	1.117	1968.2	13797.6	PZA
	*pncA*	2289222	7	V	6	2	919.0	0.700	643.5	13649.0	PZA
	*pncA*	2288820	141	Q	9	1	1425.2	0.655	934.1	11959.5	PZA
	*rpoC*	766488	1040	P	10	3	983.9	0.778	765.9	8919.5	RIF
	*pncA*	2289213	10	Q	48	3	1564.1	0.413	646.1	4897.7	PZA
	*pncA*	2288703	180	V	6	0	1762.6	0.373	657.8	4765.2	PZA
	*pncA*	2289016	76	T	5	2	798.5	0.855	682.6	4445.3	PZA
	*pncA*	2289054	63	D	6	1	1280.6	0.376	481.7	2899.9	PZA
	*pncA*	2289040	68	W	17	1	1575.1	0.598	942.5	1869.5	PZA
	*rpoA*	3878416	31	G	5	1	1200.2	0.083	99.2	253.2	.
SM	*rpsL*	781822	88	K	60	7	613.7	0.881	540.4	40005.3	SM
	*rpsL*	781687	43	K	469	11	791.2	0.632	499.9	22640.5	SM

The algorithm was run with the set of parameters that generalised best across all drugs. ‘Counts’ denotes the intermediary result of the expression in brackets in Eq 1, which is subsequently multiplied by the distance to give the initial score. In this setting, the values for *p*^1^ *g*^1^ and *p*^0^ *g*^1^ were normalised before being used in the calculations (for details see S3 Methods). SNPs from genes known to be involved in resistance to the respective drug are shaded red. SNPs from genes known to be involved in resistance to other drugs are shaded blue. Ref. AA, reference amino acid.

While runs without the *p*^1^ *g*^1^ filter and *w*_*p*^0^*g*^1^_ = 10 returned additional relevant low-frequency SNPs for INH, RIF, and PZA, they failed to produce any relevant SNPs for EMB and SM. This is a general risk of running HHS without a filter for rare alleles and normalised *p*^1^ *g*^1^ values since—given equal distance values—SNPs with *p*^1^ *g*^1^ = 2 and *p*^0^ *g*^1^ = 0 are assigned the same score as SNPs with *p*^1^ *g*^1^ = 1000 and *p*^0^ *g*^1^ = 0. Additionally, very-low-frequency SNPs are less likely to sufficiently overlap with other strong SNPs which lets them take out truly resistance-conferring variants easily.

Not normalising *p*^1^ *g*^1^ strongly favoured the most common alleles leading to a drastically reduced set of final SNPs (often only one). This is due to high-frequency co-resistant false positives not being removed quickly enough and outliving less common true positives. Setting *w*_*p*^0^*g*^1^_ to 10 partially mitigated this issue as the initial scores of false positives with non-zero *p*^0^ *g*^1^ values were substantially lower or negative.

Not accounting for intra-*p*^1^ *g*^1^ distance (i.e. setting the weight for *d*_*p*^1^*g*^1^, *i*_ to 0) had substantial influence on the results in most but not all settings. This shows that even a primitive distance metric like the average pairwise Hamming distance is a good surrogate for incorporating population structure.

**Fig 5:**
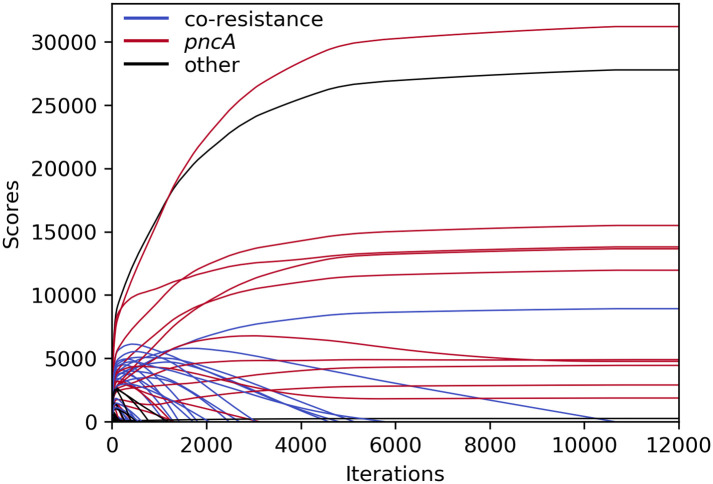
Course of HHS scores for PZA during iterative elimination. Scores remained unchanged after ∼11,000 iterations. SNPs related to PZA resistance in red; SNPs related to other drugs in blue; other SNPs in black.

An exemplary depiction of the changing scores over the course of an HHS run (with the settings used to generate the results in Table 5) is shown in Fig 5. After an initial rise, scores of co-resistant SNPs (blue) slowly decreased until they dropped below zero and the corresponding variants were purged. However, some *pncA* variants, which almost completely overlapped with the most common co-occurring resistance markers (e.g. *katG* S315 or *rpoB* S450), shared the same fate as those did not disappear quickly enough. After increasing the penalty for *p*^0^ *g*^1^ by setting *w*_*p*^0^*g*^1^_ to a larger value, co-occurring SNPs started off with lower initial scores and more *pncA* variants were retained (S5 Data). Additionally, two rare *pncA* variants (T100 and T160; occurring in eight and nine samples, respectively) overlapped in eight samples and were therefore eliminated quickly.

All SNPs shaded red or blue in Table 5 are already known to confer resistance to the respective or a different drug. For PZA, however, two variants might constitute novel discoveries and thus warrant closer examination. The *rpoA* G31 mutation started and finished with an exceptionally low score due to the samples featuring this variant being closely related (resulting in a very low average pairwise distance, S9 Fig). Additionally, it was only found in two out of the 14 different parameter configurations tested (S5 Data). Furthermore, it overlapped with two *pncA* SNPs (D8 and C138—both are marked as conferring resistance against PZA in the TBProfiler database [10]), which occurred in fewer than five samples and thus were excluded from the dataset. In light of this complementary evidence, it is reasonable to suspect *rpoA* G31 to be a false positive caused by population structure.

The five strains harbouring *hadA* C61, on the other hand, were more widely spread out across the phylogeny and the mutation has apparently emerged independently four times (S9 Fig). Moreover, it was among the final SNPs in nine out of 14 HHS runs with different parameters. However, three of the five samples carrying *hadA* C61 also featured *pncA* variants that confer resistance according to TBProfiler (T100, V139, V180). Two of these were too rare to be included in the analysis and for the third one the initial score was already below 0. Therefore, *hadA* C61 might likewise be a spurious result and its validity should be checked by including more samples or consulting orthogonal methods in future work.

## Conclusion

In a world of increasing antibiotic resistance and with the prospect of bringing routine whole-genome sequencing of pathogen samples to the clinic [56], the development of methods for predicting resistance from genomic data with near-perfect accuracy becomes a necessity. The results reported here corroborate the findings of recent studies that many ML models are well suited for phenotype prediction with equivalent or superior performance compared to traditional methods based on direct association. This might be owed to ML’s ability to learn subtle, latent interactions, which have not been discovered yet. Alternatively, some predictions may be biased by co-occurring resistant markers, causing overestimation of performance. Our results show that L1 penalised linear models are more robust in this regard and hence might be better suited for mining feature importances even though other classifiers are likely to provide more accurate predictions. Nonetheless, frequent co-occurring resistance in the dataset tested here was a major source of confounded results for all ML techniques as well as traditional GWAS approaches and tests for convergent evolution. While many strategies have been devised to account for population stratification and lineage effects in bacterial genomics, the co-occurrence issue has not received as much attention. Here, we have introduced HHS, a new method for determining resistance-associated SNPs, that tries to only allow a single variant to account for resistance in a given sample. Although potentially losing information on compensatory and cooperative mutations, the process showed great robustness towards co-occurring resistance and returned rare variants occurring in fewer than 1% of resistant samples. In spite of being strongly associated with the phenotype, these might go unnoticed by other methods due to false positives with greater significance rankings. Thus, we consider HHS a useful addition to the toolbox of bacterial genomics.

## Supporting information

S1 Fig Neural net architecture for the multidrug case. For the five single-drug equivalents the input dimensions were adjusted accordingly and the output layer only had one node.
(TIF)

S2 Fig Samples per country. Origin of samples in the dataset; more than two thirds came from the first five countries.
(TIF)

S3 Fig Distribution of all SNPs vs missense SNPs only. Kernel density plot showing the distribution of SNPs (blue) and missense SNPs (red) across the *M. tuberculosis* chromosome for all Beijing strains.
(TIF)

S4 Fig Distribution of allele count and non-calls among all SNPs and missense SNPs only. Histograms and kernel density plots (dashed red line) of allele count (top row) and missing genotypes (bottom row) for all SNPs (left column) and missense SNPs only (right column).
(TIF)

S5 Fig PCA score plot. Samples in reduced feature space of the first three principal components extracted from the missense SNPs coloured by country (left column) and resistance status (right column).
(TIF)

S6 Fig Different approaches for assessing feature importance for logistic regression with L1 (top row) and L2 (bottom row) penalties. Left column: top 20 SNPs as sorted by absolute magnitude of regression coefficients; centre column: top 20 SNPs as sorted by *p*-values calculated with approach 1; right column: top 20 SNPs as sorted by *p*-values calculated with approach 2 (for an explanation of the two approaches see the Methods section). Grey cells denote a *p*-value of 1. This means that in the L2 penalised models for EMB and PZA not a single SNP achieved a *p*-value lower than 1 according to approach 2.
(TIF)

S7 Fig Phylogenetic tree highlighting all samples with a SNP at genomic position 2238734. Resistant strains are depicted in red; susceptible strains in blue; strains with the reference genotype (i.e. the same as in H37Rv) in grey. Ref. GT, reference genotype.
(TIF)

S8 Fig Phylogenetic tree highlighting samples carrying potential novel resistance-associated variants discovered in the ML feature importances. For details regarding the variants see Table 4. Resistant strains are depicted in red; susceptible strains in blue; strains with the reference genotype (i.e. the same as in H37Rv) in grey. Ref. GT, reference genotype.
(TIF)

S9 Fig Phylogenetic tree highlighting samples with potential novel resistance-related SNPs discovered by HHS. All samples with *rpoA* G31 (genomic position 3878416) sit on the same clade, whereas those featuring *hadA* C61 (genomic position 732110) occur in four distinct locations throughout the tree.
(TIF)

S1 Table Results of *pyseer*’s SEER implementation run with --max-dimensions 4. Note that the distance values are slightly different from those displayed in Table 5. This is because HHS calculates the average pairwise distance among all strains with *p*^1^ *g*^1^, whereas for the distances listed in the Supplementary Tables all the strains in the dataset have been used.
(PDF)

S2 Table Results of *pyseer*’s SEER implementation run with --max-dimensions 10. Note that the distance values are slightly different from those displayed in Table 5. This is because HHS calculates the average pairwise distance among all strains with *p*^1^ *g*^1^, whereas for the distances listed in the Supplementary Tables all the strains in the dataset have been used.
(PDF)

S3 Table Significant SNPs detected by *treeWAS*. *p*-values were calculated by *treeWAS* internally and all rounded to zero by R before returning the result (R prints six decimal places by default). Hence, they have been omitted in the table. *treeWAS* scores with insignificant *p*-values are replaced by ‘ins.’. Note that the distance values are slightly different from those displayed in Table 5. This is because HHS calculates the average pairwise distance among all strains with *p*^1^ *g*^1^, whereas for the distances listed in the Supplementary Tables all the strains in the dataset have been used.
(PDF)

S4 Table Most important SNPs for ML predictions that are not (yet) known to be related to resistance. For every drug the top 20 SNPs with the greatest feature importances were extracted and those which were associated with any drug in the TBProfiler database [10] were dropped. The others represent potentially novel resistance markers and are listed here. Duplicated rows from SNPs that were important for more than one ML model were removed. Depending on the ML algorithm, feature importance was quantified via different metrics. For Lasso and SVM the absolute magnitudes of the regression coefficients were used (the higher, the more important). *p*-values for Logistic regression were calculated with approach 1 (the lower, the more important). Gini importances (i.e. normalised total reduction of the Gini impurity [57]; the higher, the more important) for the tree-based classifiers were calculated by the *sklearn* implementations internally. The last nine columns show how the respective SNP ranked in importance for the single ML methods. Note, that for most models SNPs that were already known (and that were therefore removed) scored highest. Since they were excluded (and due to dropped duplicated rows), not all ranks from one to 20 are present. Ranks higher than 999 have been replaced with a ‘+’ sign. For unknown genes the corresponding locus tag has been placed into the ‘Gene’ column. AA, amino acid; Dist., distance; FI, feature importance; coef., coefficient; *p*-val., *p*-value; GI, Gini importance. For other abbreviations see Fig 3.
(PDF)

S1 Methods Command line arguments used in the phylogenomic analysis and GWAS. (TXT)

S2 Methods *sklearn* grid search and model parameters. (TXT)

S3 Methods HHS algorithm and parameters. (PDF)

S1 Data GWAS results (SEER + LMM combined). Top 20 most significant SNPs per drug and GWAS implementation.
(ODS)

S2 Data GWAS LMM results with lineage effects (*bugwas*). Top 20 most significant SNPs per lineage and drug. The five single-drug datasets contained different SNPs. Thus, for the different drugs different lineages were found and some did not contain any significant variants.
(ODS)

S3 Data ML feature importances. Top 20 SNPs per ML algorithm and drug.
(ODS)

S4 Data Logistic regression feature importances. Magnitudes of regression coefficients as well as *p*-values calculated by approaches 1 and 2.
(ODS)

S5 Data HHS results for all parameter combinations tested. (ODS)

S6 Data Strain metadata. (CSV)

S7 Data Filtered genotypes. (TAR.GZ)
