# Supplementary figures and images for: Robust detection of point mutations involved in multidrug-resistant Mycobacterium tuberculosis in the presence of co-occurrent resistance markers
Source: PLoS Comput Biol. 2020 Dec 21;16(12):e1008518. doi: 10.1371/journal.pcbi.1008518 (PMC7785249; doi:10.1371/journal.pcbi.1008518)

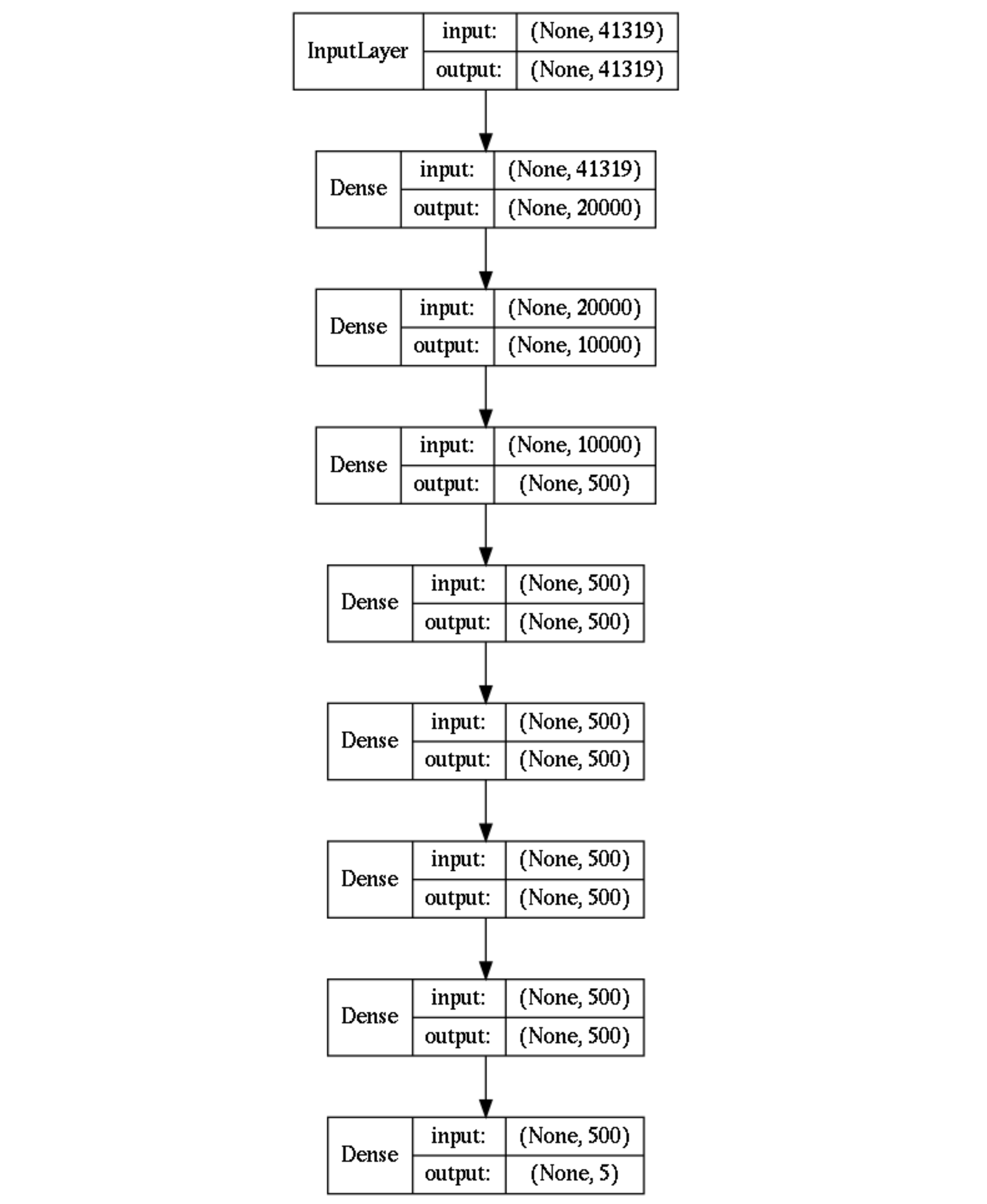

Supplement: S1 Fig — For the five single-drug equivalents the input dimensions were adjusted accordingly and the output layer only had one node. (TIF) [file pcbi.1008518.s001.tif]

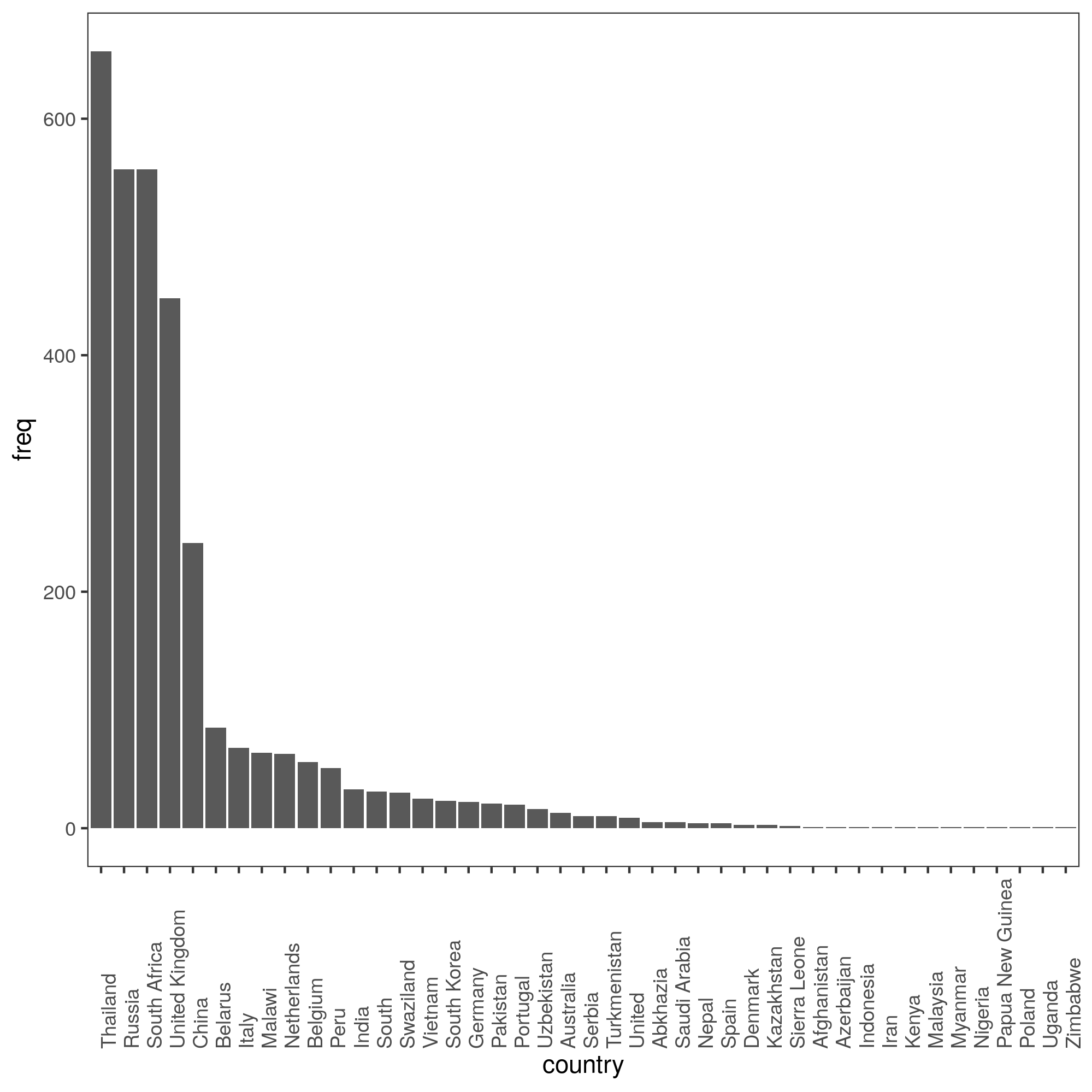

Supplement: S2 Fig — Origin of samples in the dataset; more than two thirds came from the first five countries. (TIF) [file pcbi.1008518.s002.tif]

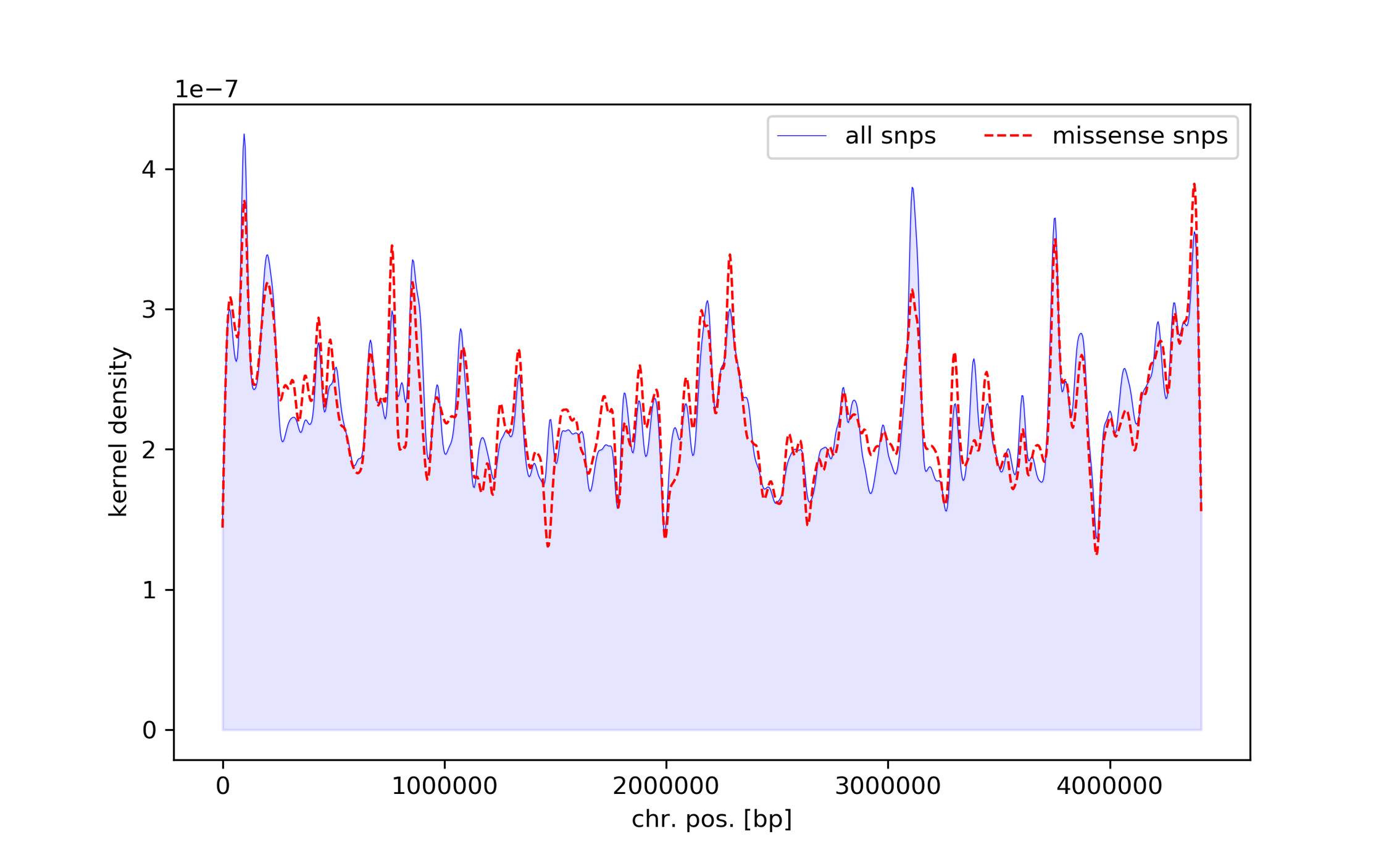

Supplement: S3 Fig — Kernel density plot showing the distribution of SNPs (blue) and missense SNPs (red) across the M. tuberculosis chromosome for all Beijing strains. (TIF) [file pcbi.1008518.s003.tif]

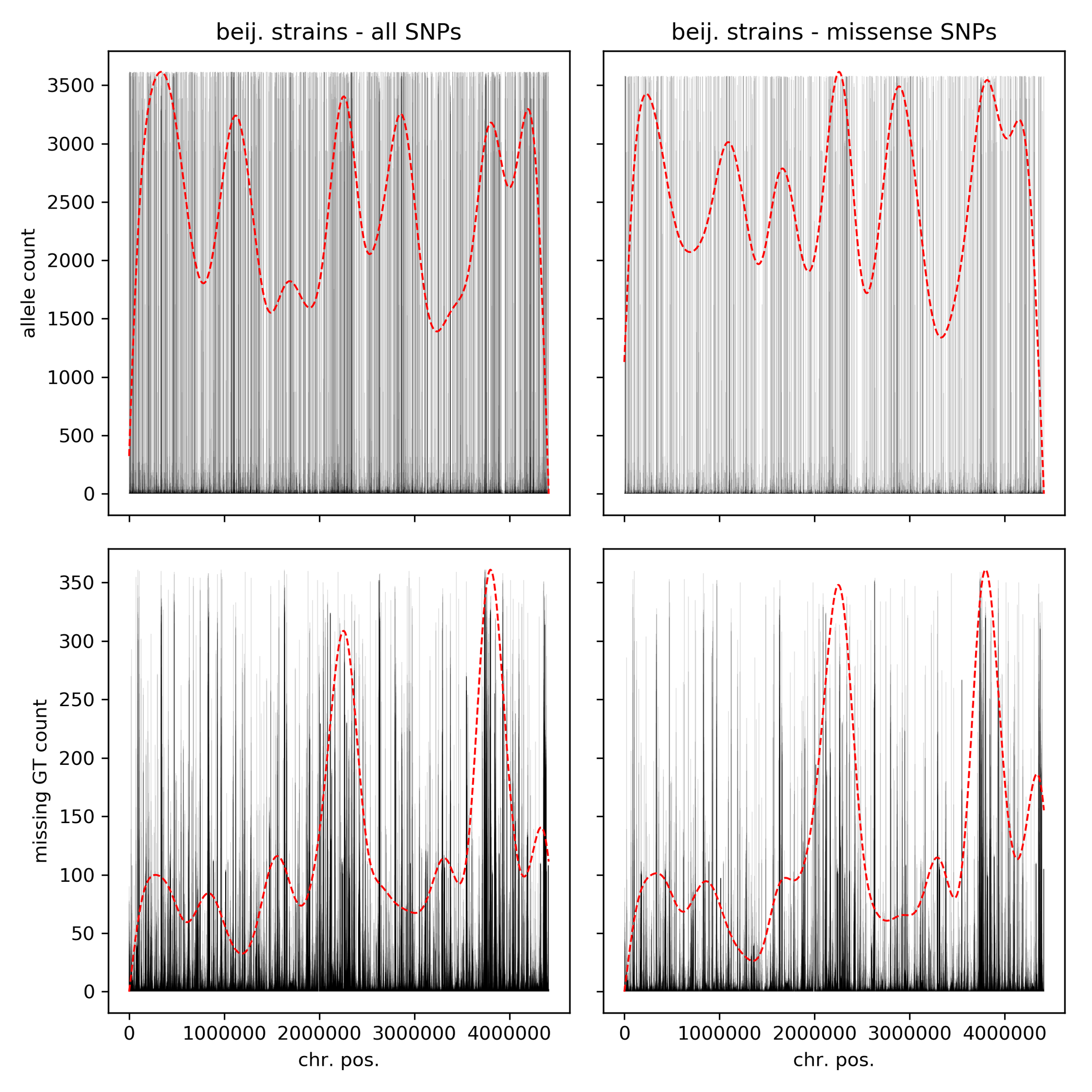

Supplement: S4 Fig — Histograms and kernel density plots (dashed red line) of allele count (top row) and missing genotypes (bottom row) for all SNPs (left column) and missense SNPs only (right column). (TIF) [file pcbi.1008518.s004.tif]

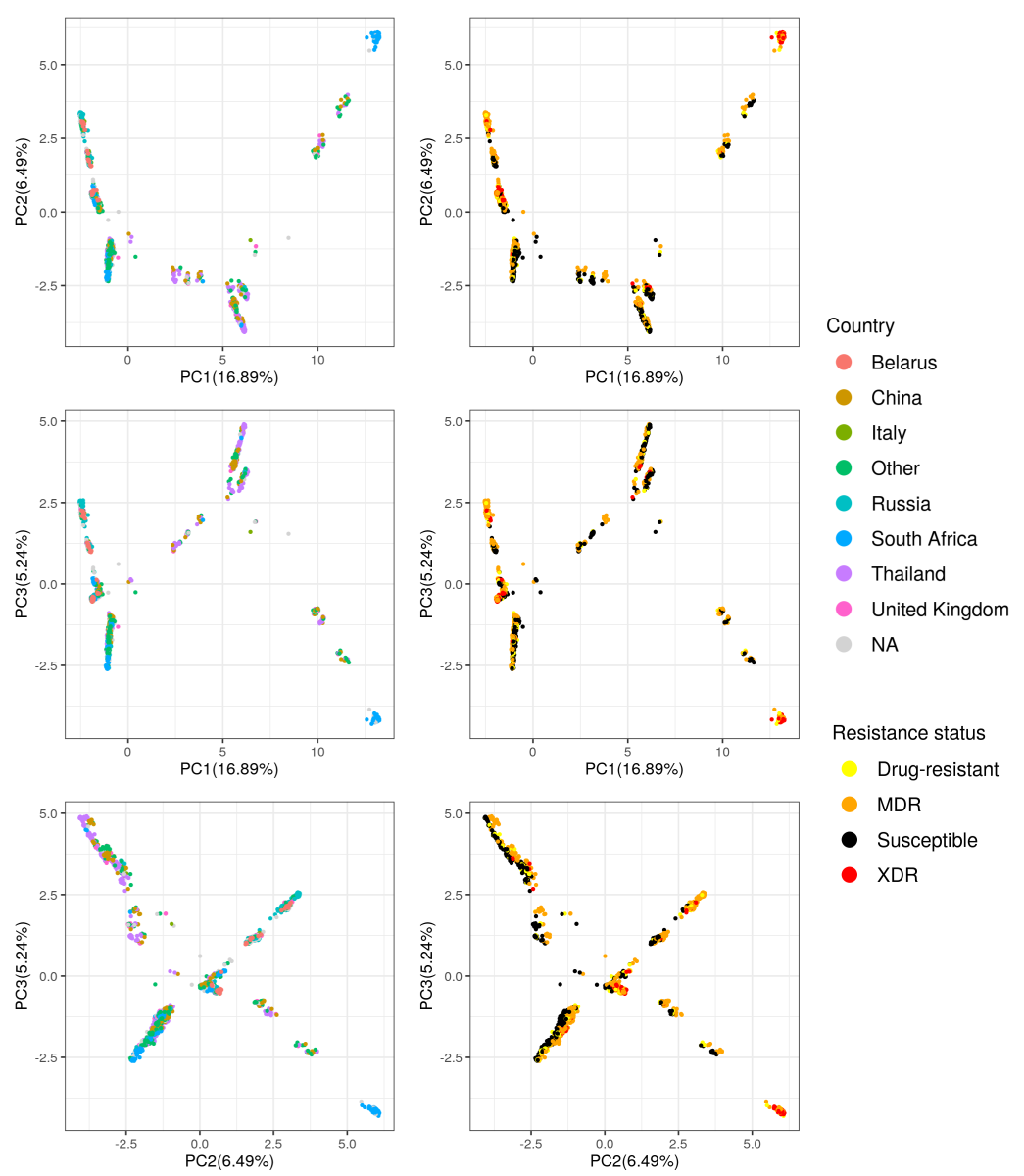

Supplement: S5 Fig — Samples in reduced feature space of the first three principal components extracted from the missense SNPs coloured by country (left column) and resistance status (right column). (TIF) [file pcbi.1008518.s005.tif]

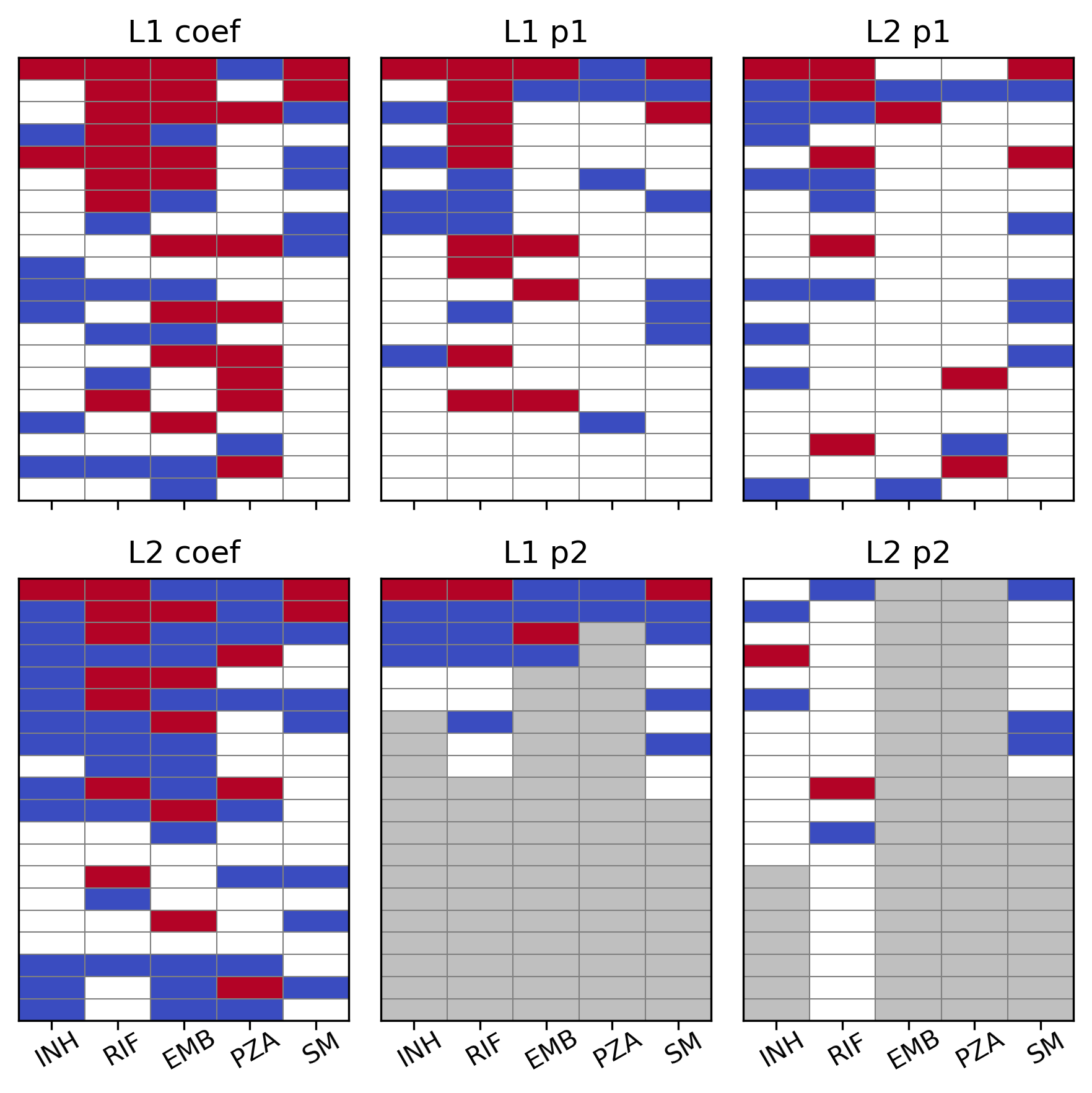

Supplement: S6 Fig — Left column: top 20 SNPs as sorted by absolute magnitude of regression coefficients; centre column: top 20 SNPs as sorted by p-values calculated with approach 1; right column: top 20 SNPs as sorted by p-values calculated with approach 2 (for an explanation of the two approaches see the Methods section). Grey cells denote a p-value of 1. This means that in the L2 penalised models for EMB and PZA not a single SNP achieved a p-value lower than 1 according to approach 2. (TIF) [file pcbi.1008518.s006.tif]

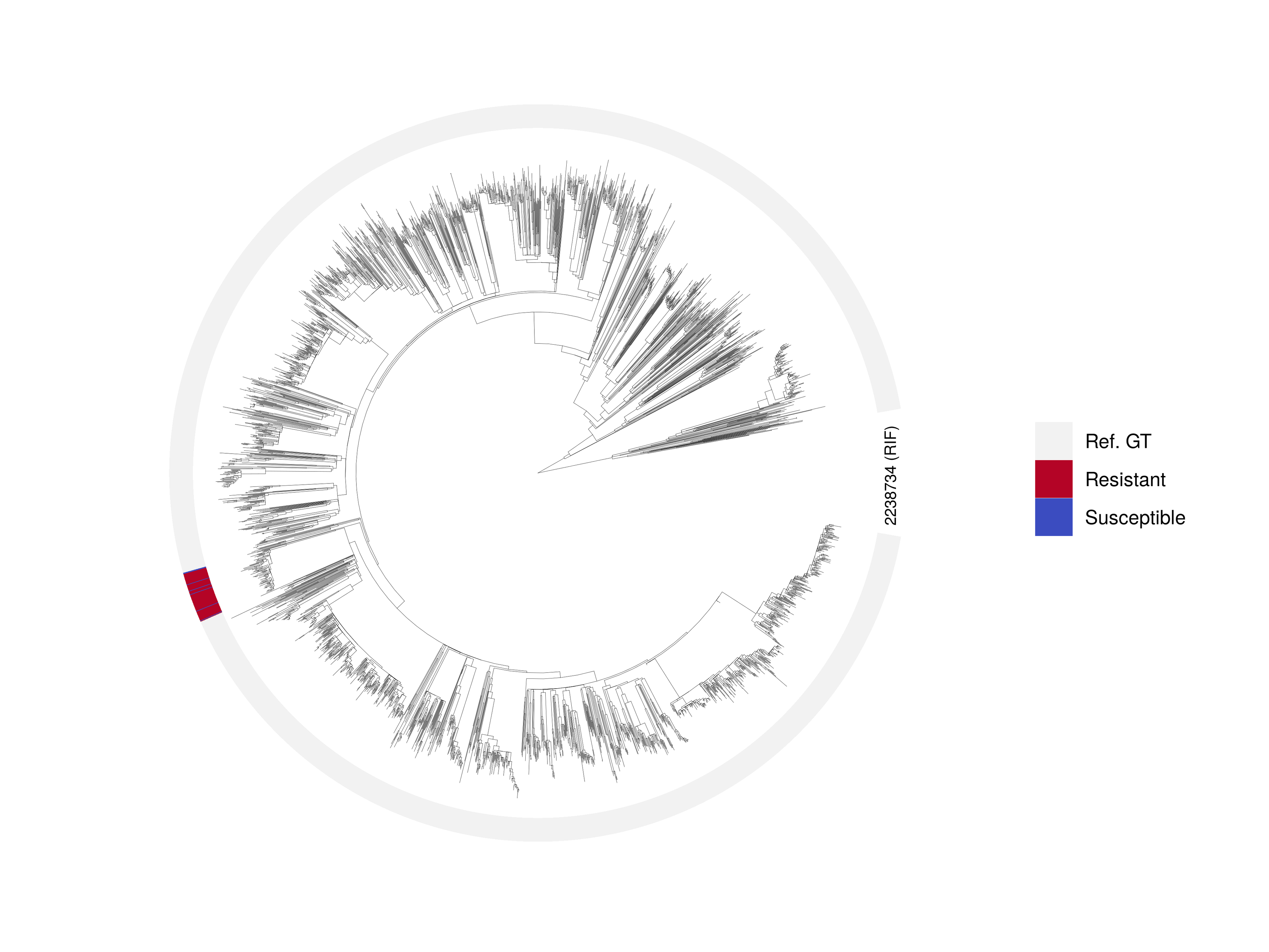

Supplement: S7 Fig — Resistant strains are depicted in red; susceptible strains in blue; strains with the reference genotype (i.e. the same as in H37Rv) in grey. Ref. GT, reference genotype. (TIF) [file pcbi.1008518.s007.tif]

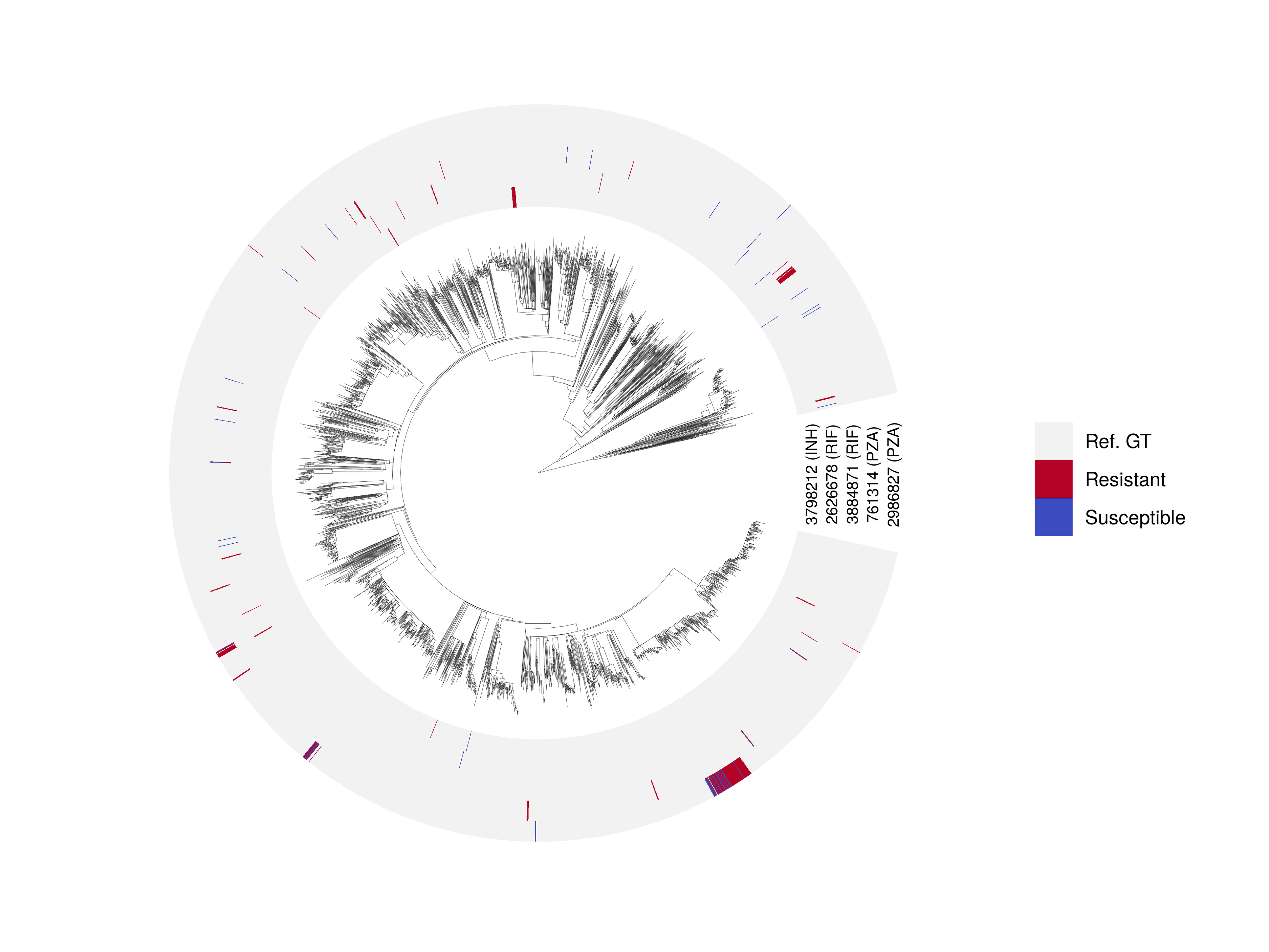

Supplement: S8 Fig — For details regarding the variants see Table 4. Resistant strains are depicted in red; susceptible strains in blue; strains with the reference genotype (i.e. the same as in H37Rv) in grey. Ref. GT, reference genotype. (TIF) [file pcbi.1008518.s008.tif]

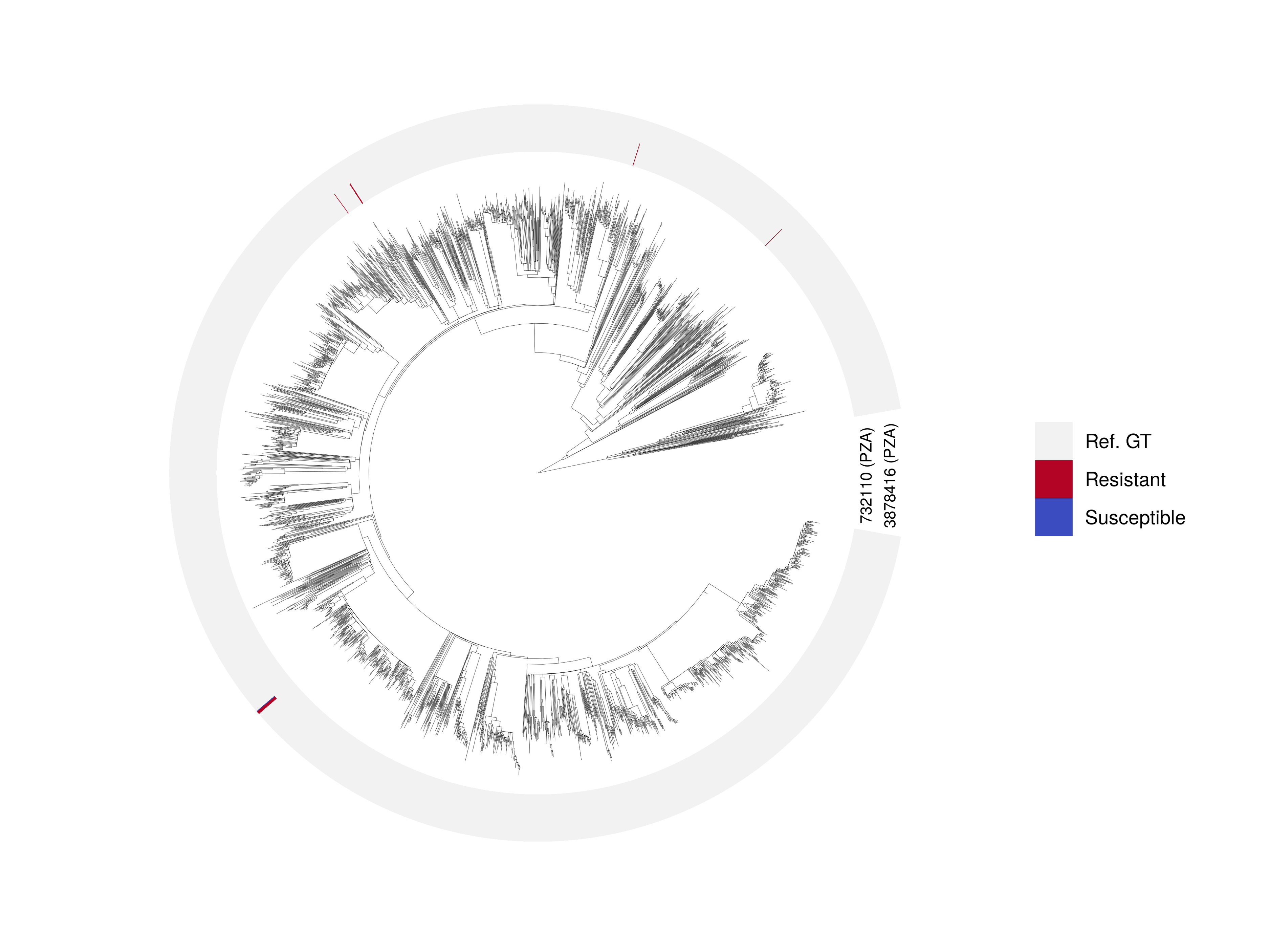

Supplement: S9 Fig — All samples with rpoA G31 (genomic position 3878416) sit on the same clade, whereas those featuring hadA C61 (genomic position 732110) occur in four distinct locations throughout the tree. (TIF) [file pcbi.1008518.s009.tif]
