# Supplementary material for: Robust detection of point mutations involved in multidrug-resistant Mycobacterium tuberculosis in the presence of co-occurrent resistance markers
Source: PLoS Comput Biol. 2020 Dec 21;16(12):e1008518. doi: 10.1371/journal.pcbi.1008518 (PMC7785249; doi:10.1371/journal.pcbi.1008518)
Supplement: S1 Table — Note that the distance values are slightly different from those displayed in Table 5. This is because HHS calculates the average pairwise distance among all strains with p1 g1, whereas for the distances listed in the Supplementary Tables all the strains in the dataset have been used. (PDF) [file pcbi.1008518.s010.pdf]

S1 Table. ***pyseer*'s SEER implementation run with --max-dimensions 4**

Drug	Position	Gene	Codon	Ref. AA	p^1g^1	p^0g^1	Distance	p-value	Resistance
INH	2155168	*katG*	315	S	1676	12	0.964	0	INH
	761155	*rpoB*	450	S	1289	54	0.847	1.5e-257	RIF
	781687	*rpsL*	43	K	1118	79	0.768	2.5e-228	SM
	4247429	*embB*	306	M	583	24	0.804	2.3e-103	EMB
RIF	761155	*rpoB*	450	S	1367	9	0.847	0	RIF
	2155168	*katG*	315	S	1548	169	0.964	0	INH
	781687	*rpsL*	43	K	1044	162	0.768	6.0e-161	SM
	4247429	*embB*	306	M	597	24	0.804	6.9e-124	EMB
EMB	2155168	*katG*	315	S	1004	587	0.964	1.3e-212	INH
	761155	*rpoB*	450	S	854	433	0.847	7.8e-152	RIF
	781687	*rpsL*	43	K	729	381	0.768	9.7e-127	SM
	4247429	*embB*	306	M	455	120	0.804	1.5e-104	EMB
PZA	2155168	*katG*	315	S	623	558	0.964	1.4e-80	INH
	761155	*rpoB*	450	S	549	452	0.847	9.2e-76	RIF
	4247429	*embB*	306	M	292	160	0.804	1.1e-55	EMB
	781687	*rpsL*	43	K	446	378	0.768	2.2e-49	SM
SM	781687	*rpsL*	43	K	469	11	0.768	3.7e-152	SM
	2155168	*katG*	315	S	542	80	0.964	1.0e-103	INH
	761155	*rpoB*	450	S	411	74	0.847	5.9e-43	RIF
	4338371	*whiB6*	51	T	100	0	0.183	7.4e-31	.
