# Supplementary material for: Robust detection of point mutations involved in multidrug-resistant Mycobacterium tuberculosis in the presence of co-occurrent resistance markers
Source: PLoS Comput Biol. 2020 Dec 21;16(12):e1008518. doi: 10.1371/journal.pcbi.1008518 (PMC7785249; doi:10.1371/journal.pcbi.1008518)
Supplement: S2 Table — Note that the distance values are slightly different from those displayed in Table 5. This is because HHS calculates the average pairwise distance among all strains with p1 g1, whereas for the distances listed in the Supplementary Tables all the strains in the dataset have been used. (PDF) [file pcbi.1008518.s011.pdf]

**S1 Table. *pyseer*'s SEER implementation run with --max-dimensions 10**

| Drug | Position | Gene | Codon | Ref. AA | $p^1g^1$ | $p^0g^1$ | Distance | $p$-value | Resistance |
|---|---|---|---|---|---|---|---|---|---|
| INH | 2155168 | *katG* | 315 | S | 1676 | 12 | 0.964 | 0 | INH |
| | 761155 | *rpoB* | 450 | S | 1289 | 54 | 0.847 | 3.9e-192 | RIF |
| | 781687 | *rpsL* | 43 | K | 1118 | 79 | 0.768 | 1.3e-155 | SM |
| | 4247429 | *embB* | 306 | M | 583 | 24 | 0.804 | 4.6e-91 | EMB |
| RIF | 761155 | *rpoB* | 450 | S | 1367 | 9 | 0.847 | 0 | RIF |
| | 2155168 | *katG* | 315 | S | 1548 | 169 | 0.964 | 1.4e-260 | INH |
| | 781687 | *rpsL* | 43 | K | 1044 | 162 | 0.768 | 7.0e-138 | SM |
| | 4247429 | *embB* | 306 | M | 597 | 24 | 0.804 | 1.3e-124 | EMB |
| EMB | 2155168 | *katG* | 315 | S | 1004 | 587 | 0.964 | 5.2e-207 | INH |
| | 761155 | *rpoB* | 450 | S | 854 | 433 | 0.847 | 3.5e-154 | RIF |
| | 781687 | *rpsL* | 43 | K | 729 | 381 | 0.768 | 7.2e-137 | SM |
| | 4247429 | *embB* | 306 | M | 455 | 120 | 0.804 | 1.1e-103 | EMB |
| PZA | 2155168 | *katG* | 315 | S | 623 | 558 | 0.964 | 2.2e-85 | INH |
| | 761155 | *rpoB* | 450 | S | 549 | 452 | 0.847 | 4.3e-66 | RIF |
| | 4247429 | *embB* | 306 | M | 292 | 160 | 0.804 | 8.0e-41 | EMB |
| | 781687 | *rpsL* | 43 | K | 446 | 378 | 0.768 | 3.2e-31 | SM |
| SM | 781687 | *rpsL* | 43 | K | 469 | 11 | 0.768 | 1.1e-138 | SM |
| | 2155168 | *katG* | 315 | S | 542 | 80 | 0.964 | 2.7e-85 | INH |
| | 761155 | *rpoB* | 450 | S | 411 | 74 | 0.847 | 2.3e-30 | RIF |
| | 1722228 | *pks5* | 2061 | L | 656 | 826 | 1.129 | 1.1e-29 | . |
