# Supplementary material for: Robust detection of point mutations involved in multidrug-resistant Mycobacterium tuberculosis in the presence of co-occurrent resistance markers
Source: PLoS Comput Biol. 2020 Dec 21;16(12):e1008518. doi: 10.1371/journal.pcbi.1008518 (PMC7785249; doi:10.1371/journal.pcbi.1008518)
Supplement: S3 Table — p-values were calculated by treeWAS internally and all rounded to zero by R before returning the result (R prints six decimal places by default). Hence, they have been omitted in the table. treeWAS scores with insignificant p-values are replaced by ‘ins.’. Note that the distance values are slightly different from those displayed in Table 5. This is because HHS calculates the average pairwise distance among all strains with p1 g1, whereas for the distances listed in the Supplementary Tables all the strains in the dataset have been used. (PDF) [file pcbi.1008518.s012.pdf]

S3 Table. Significant SNPs detected by *treeWAS*.

Drug	Position	Gene	Codon number	Ref. AA	p^1g^1	p^0g^1	Distance	Score 1	Score 2	Score 3	Resistance
INH	761155	*rpoB*	450	S	1289	54	0.847	0.600	79	4238.3	RIF
	781687	*rpsL*	43	K	1118	79	0.768	0.487	83	3516.7	SM
	2155168	*katG*	315	S	1676	12	0.964	0.843	157	5728.3	INH
	4247429	*embB*	306	M	583	24	0.804	ins.	37	ins.	EMB
RIF	166565	.	247	I	1116	491	0.569	0.296	ins.	ins.	.
	484005	*fadD30*	10	R	1115	491	0.569	0.295	ins.	ins.	.
	761139	*rpoB*	445	H	135	4	1.078	ins.	35	ins.	RIF
	761155	*rpoB*	450	S	1367	9	0.847	0.714	134	5095.7	RIF
	781687	*rpsL*	43	K	1044	162	0.768	0.445	85	3418.3	SM
	941722	.	178	I	1115	490	0.569	0.297	ins.	ins.	.
	1151304	*kdpD*	128	R	1115	491	0.569	0.295	ins.	ins.	.
	1446733	*argS*	119	A	1115	492	0.569	0.296	ins.	ins.	.
	2155168	*katG*	315	S	1548	169	0.964	0.723	121	5238.3	INH
	2838897	.	215	M	1115	490	0.569	0.296	ins.	ins.	.
	3056767	.	156	P	1113	489	0.569	0.296	ins.	ins.	.
	4046007	.	38	V	1119	501	0.574	0.292	ins.	ins.	.
	4215433	.	115	A	1115	491	0.569	0.296	ins.	ins.	.
	4247429	*embB*	306	M	597	24	0.804	ins.	52	ins.	EMB
EMB	7582	*gyrA*	94	D	203	83	1.150	ins.	27	ins.	FQ
	761155	*rpoB*	450	S	854	433	0.847	0.586	79	4325.3	RIF
	781687	*rpsL*	43	K	729	381	0.768	0.544	64	4092.3	SM
	2155168	*katG*	315	S	1004	587	0.964	0.583	86	4325.7	INH
	4247429	*embB*	306	M	455	120	0.804	0.536	63	ins.	EMB
PZA	761155	*rpoB*	450	S	549	452	0.847	ins.	36	ins.	RIF
	781687	*rpsL*	43	K	446	378	0.768	ins.	24	ins.	SM
	2155168	*katG*	315	S	623	558	0.964	ins.	40	ins.	INH
	4247429	*embB*	306	M	292	160	0.804	ins.	25	ins.	EMB
SM	761155	*rpoB*	450	S	411	74	0.847	ins.	33	ins.	RIF
	781687	*rpsL*	43	K	469	11	0.768	0.731	78	2112.0	SM
	781822	*rpsL*	88	K	60	7	0.971	ins.	19	ins.	SM
	2155168	*katG*	315	S	542	80	0.964	0.736	65	2265.7	INH
	2635577	*PPE39*	6	R	160	239	1.071	ins.	23	ins.	.
	4247429	*embB*	306	M	184	40	0.804	ins.	20	ins.	EMB
