# Supplementary material for: Robust detection of point mutations involved in multidrug-resistant Mycobacterium tuberculosis in the presence of co-occurrent resistance markers
Source: PLoS Comput Biol. 2020 Dec 21;16(12):e1008518. doi: 10.1371/journal.pcbi.1008518 (PMC7785249; doi:10.1371/journal.pcbi.1008518)
Supplement: S4 Table — For every drug the top 20 SNPs with the greatest feature importances were extracted and those which were associated with any drug in the TBProfiler database [10] were dropped. The others represent potentially novel resistance markers and are listed here. Duplicated rows from SNPs that were important for more than one ML model were removed. Depending on the ML algorithm, feature importance was quantified via different metrics. For Lasso and SVM the absolute magnitudes of the regression coefficients were used (the higher, the more important). p-values for Logistic regression were calculated with approach 1 (the lower, the more important). Gini importances (i.e. normalised total reduction of the Gini impurity [57]; the higher, the more important) for the tree-based classifiers were calculated by the sklearn implementations internally. The last nine columns show how the respective SNP ranked in importance for the single ML methods. Note, that for most models SNPs that were already known (and that were therefore removed) scored highest. Since they were excluded (and due to dropped duplicated rows), not all ranks from one to 20 are present. Ranks higher than 999 have been replaced with a ‘+’ sign. For unknown genes the corresponding locus tag has been placed into the ‘Gene’ column. AA, amino acid; Dist., distance; FI, feature importance; coef., coefficient; p-val., p-value; GI, Gini importance. For other abbreviations see Fig 3. (PDF) [file pcbi.1008518.s013.pdf]

S4 Table. ML feature importance of yet unknown variants for INH

Method	Position	Gene (Locus Tag)	Ref. AA	Codon	p^1g^1	p^0g^1	Dist.	FI value	Rank								
									Lasso	LR-L1	LR-L2	SVM-L1	SVM-L2	DT	DT-MD5	RF	GBM
Lasso (coef.)	1571402	(Rv1395)	D	119	6	1	0.116	0.526	3	20	36	3	14	11	+	8	39
	3732118	*PPE54*	F	1606	13	0	0.032	0.503	4	213	98	29	32	+	+	35	+
	1920155	*recN*	A	158	2	0	0.012	0.353	6	16	26	8	44	21	+	+	98
	3004785	(Rv2688c)	D	289	39	29	0.089	0.310	8	33	38	59	328	+	+	405	+
	707790	(Rv0613c)	G	576	3	3	0.054	0.308	9	50	48	123	46	+	+	53	532
	3393551	(Rv3033)	P	58	4	0	0.408	0.283	11	+	685	+	298	+	+	390	+
	2445452	(Rv2183c)	S	120	2	0	0.930	0.274	12	240	+	+	539	+	+	+	+
	2717664	*gpgP*	G	46	2	2	0.044	0.273	13	78	93	16	100	35	+	+	585
	1030058	(Rv0923c)	T	174	42	16	0.463	0.197	17	69	54	64	27	+	+	275	+
	2981070	(Rv2660c)	V	41	3	5	0.035	0.197	18	18	24	15	26	92	+	17	28
	3741399	*PE_PGRS50*	V	459	3	4	0.517	0.193	20	24	353	33	376	+	+	123	+
LR-L1 (p-val.)	2300546	*pks12*	H	2147	1921	1540	1.000	3e-157	26	2	27	4	39	37	+	444	339
	3842620	*PPE57*	T	128	1933	1517	0.997	1e-21	61	4	12	+	140	+	+	+	+
	1165114	(Rv1042c)	G	129	1762	1363	0.969	9e-16	109	6	5	66	22	+	+	509	703
	1789565	(Rv1588c)	R	91	1816	1437	0.884	2e-07	128	9	+	51	148	+	+	+	289
	2028992	*PPE27*	P	190	42	37	0.279	8e-07	87	10	+	86	+	+	+	528	+
	3883915	(Rv3466)	V	131	194	530	1.006	1e-06	68	11	47	45	21	8	7	5	7
	1998063	(Rv1765c)	E	151	1333	962	1.020	7e-06	148	12	7	81	43	+	+	32	180
	2550014	(Rv2277c)	T	6	280	168	1.158	3e-05	76	13	9	52	10	80	+	39	212
	908186	*sseC2*	T	100	1766	1459	0.870	9e-05	+	15	8	112	152	+	+	+	+
	2201720	(Rv1954c)	T	9	29	29	0.146	4e-04	59	17	33	85	15	+	+	+	+
	2262620	(Rv2015c)	E	151	1539	1057	1.002	5e-04	156	19	10	90	102	+	+	49	227
LR-L2 (p-val.)	2295685	*pks12*	V	3768	1845	1457	0.982	0.040	113	+	14	+	136	+	+	178	833
	2630740	*plcA*	T	446	1893	1485	0.991	0.046	+	+	16	+	926	+	+	+	+
	3135912	(Rv2828c)	T	141	1632	1276	0.769	0.063	+	181	17	+	504	+	+	+	+
	3883796	(Rv3466)	R	91	1683	1274	0.759	0.081	135	+	18	+	198	+	+	+	+
	3135951	(Rv2828c)	S	128	1643	1280	0.766	0.098	+	32	19	+	754	+	+	+	+
SVM-L1 (coef.)	1778561	(Rv1571)	V	8	14	0	0.032	0.571	+	209	96	9	33	+	+	34	18
	2762364	(Rv2459)	R	504	7	3	0.278	0.495	+	49	46	11	47	+	+	52	550
	2741336	*dctA*	W	210	8	0	0.092	0.468	+	434	522	12	221	+	+	12	+
	4097010	(Rv3659c)	W	329	28	28	0.077	-0.346	48	22	34	18	16	+	+	453	+
	3798212	*idsB*	P	93	12	2	0.688	0.340	22	63	264	19	66	+	+	79	284
SVM-L2 (coef.)	413268	*iniC*	V	171	0	13	0.037	-0.077	37	111	35	54	19	+	+	98	+
DT (GI)	1657327	*ctpD*	A	122	1	0	NA	0.013	+	+	+	+	+	4	+	14	13
	3732122	*PPE54*	A	1605	13	0	0.032	9e-03	51	210	97	34	31	6	+	26	454
	759916	*rpoB*	K	37	8	0	0.092	8e-03	+	466	525	+	223	7	+	28	119
	2541952	(Rv2267c)	L	287	11	78	0.154	5e-03	+	+	+	+	+	9	8	111	37
	3005631	(Rv2688c)	A	7	0	1	NA	4e-03	+	+	+	+	+	12	9	+	10
	4095721	(Rv3657c)	V	132	0	1	NA	3e-03	+	+	+	+	+	13	+	75	15
	2958814	(Rv2631)	S	415	0	1	NA	3e-03	+	+	+	+	+	14	+	+	14
	3538266	(Rv3169)	D	343	1	0	NA	3e-03	+	759	+	+	+	16	+	+	16
	374310	*PPE6*	P	468	0	1	NA	3e-03	+	+	+	+	+	17	+	+	11
	3732408	*PPE54*	T	1510	1	0	NA	3e-03	+	+	+	+	844	18	+	+	20
	2099088	*ureC*	E	376	1	0	NA	3e-03	+	+	+	+	+	19	+	+	331
	1620698	*bisC*	A	303	2	0	0.836	3e-03	+	+	+	+	+	20	+	+	55
DT-MD5 (GI)	1657335	*ctpD*	G	125	0	1	NA	0.015	+	+	+	+	+	+	4	16	241
	105675	*PPE1*	I	118	14	0	0.032	0.010	+	220	100	+	35	+	6	43	32
	2635577	*PPE39*	R	6	512	392	1.071	3e-03	166	182	+	116	+	24	10	22	57
	1701805	(Rv1510)	T	171	0	2	0.019	2e-03	+	+	+	+	+	+	11	+	591
	1483457	(Rv1320c)	M	254	0	1	NA	2e-03	+	338	412	+	365	28	12	+	215
	1677741	(Rv1488)	E	115	0	1	NA	2e-03	+	127	77	+	115	+	13	+	509
	2412676	*murG*	P	226	1	0	NA	2e-03	+	+	+	+	+	+	14	+	+
	3897155	(Rv3479)	F	446	110	74	0.503	1e-03	+	+	+	121	+	+	15	+	+
RF (GI)	4233356	(Rv3786c)	A	81	1	0	NA	2e-03	+	+	+	+	+	+	+	9	332
	1658284	*ctpD*	L	441	3	1	0.060	2e-03	+	+	+	+	+	+	+	10	58
	1658773	*ctpD*	A	604	0	1	NA	1e-03	+	+	+	+	+	+	+	18	838
	2871497	*aroE*	F	173	11	68	0.164	1e-03	+	+	211	+	+	+	+	19	86
	1657777	*ctpD*	A	272	0	1	NA	1e-03	+	+	+	+	+	+	+	20	8
GBM (GI)	1349206	(Rv1205)	V	163	8	0	0.092	3e-03	+	480	541	+	240	+	+	236	12
	3782567	*dnaE2*	R	725	1	0	NA	2e-03	+	+	160	+	287	+	+	+	19

S4 Table (continued). ML feature importance of yet unknown variants for RIF

Method	Position	Gene (Locus Tag)	Ref. AA	Codon	p^1g^1	p^0g^1	Dist.	FI value	Rank								
									Lasso	LR-L1	LR-L2	SVM-L1	SVM-L2	DT	DT-MD5	RF	GBM
Lasso (coef.)	1530714	(Rv1359)	M	181	4	0	0.041	0.276	10	101	157	24	50	+	+	239	37
	3392965	(Rv3032A)	A	52	4	0	0.077	0.271	11	83	368	32	184	+	+	+	+
	1056433	*pgi*	F	85	1	2	0.029	-0.233	13	78	111	139	28	+	8	56	23
	3294519	*pks1*	G	612	10	13	0.144	0.221	14	24	28	20	10	+	+	62	584
	988034	(Rv0888)	D	268	2	0	0.025	0.213	16	150	251	55	138	+	+	+	+
	3003147	*arsB1*	A	389	7	4	0.082	-0.211	17	17	36	11	14	+	+	49	101
	849568	*PPE12*	A	158	5	0	0.054	0.179	20	48	162	9	55	+	+	115	820
LR-L1 (p-val.)	2262620	(Rv2015c)	E	151	1485	1138	1.002	1e-08	66	11	4	83	26	+	+	75	498
	3883796	(Rv3466)	R	91	1601	1372	0.759	1e-06	107	13	+	118	+	+	+	18	80
	177857	*PE1*	L	485	1869	1670	1.001	7e-06	+	15	87	10	+	+	+	+	137
	2295685	*pks12*	V	3768	1773	1574	0.982	9e-05	72	18	32	88	729	+	+	48	+
	1218896	*PE_PGRS22*	G	810	1428	1174	0.716	1e-04	137	19	19	136	+	+	+	21	+
	2302033	*pks12*	R	1652	1826	1629	1.002	2e-04	+	20	+	78	+	+	+	+	+
LR-L2 (p-val.)	2630740	*plcA*	T	446	1832	1594	0.991	1e-04	+	62	8	106	80	22	+	698	91
	3135951	(Rv2828c)	S	128	1563	1380	0.766	5e-03	+	+	10	+	352	+	+	+	+
	3883915	(Rv3466)	V	131	196	531	1.006	6e-03	151	+	12	+	61	+	+	395	198
	1278278	(Rv1150)	D	4	1438	1123	0.919	9e-03	120	43	13	127	417	+	+	40	427
	908186	*sseC2*	T	100	1673	1570	0.870	0.015	+	59	14	+	716	+	+	337	+
	4274541	*pirG*	P	268	627	235	0.438	0.026	+	29	15	98	206	+	+	327	873
	3135912	(Rv2828c)	T	141	1555	1374	0.769	0.026	+	+	16	+	+	+	+	+	361
	3798095	*idsB*	V	132	1868	1640	1.000	0.027	+	+	17	89	365	+	+	+	114
	1286766	*mutT2*	G	58	1580	1367	0.730	0.050	+	+	20	+	+	+	+	+	+
SVM-L1 (coef.)	114853	*nrp*	A	1618	3	1	0.932	0.621	24	58	+	13	+	+	+	+	+
	3798212	*idsB*	P	93	8	6	0.688	0.509	22	25	145	17	222	+	+	164	811
	2626678	(Rv2348c)	I	101	10	4	1.484	0.499	38	49	67	18	30	19	+	20	16
	2127102	(Rv1877)	N	400	7	3	0.111	-0.489	+	31	107	19	56	+	+	+	82
SVM-L2 (coef.)	3735677	*PPE54*	V	420	12	23	1.315	0.204	33	51	46	53	17	+	+	+	+
	3884871	(Rv3467)	P	303	30	15	1.172	0.200	130	80	51	111	19	+	+	+	+
	4146860	(Rv3703c)	L	11	1	0	NA	0.194	+	68	56	68	20	+	+	+	+
DT (GI)	2238734	(Rv1995)	F	198	78	7	0.089	9e-03	+	+	+	+	+	7	+	72	262
	3740016	*PE_PGRS50*	G	920	0	1	NA	4e-03	+	+	+	+	+	15	+	759	+
	3072579	*hsdS*	G	21	0	1	NA	3e-03	+	+	+	+	+	16	+	427	386
	2635577	*PPE39*	R	6	473	433	1.071	3e-03	132	446	+	+	+	17	+	81	368
	3056767	(Rv2743c)	P	156	1113	489	0.569	3e-03	+	+	+	+	+	18	+	15	84
	374310	*PPE6*	P	468	0	1	NA	3e-03	+	+	+	+	+	20	+	26	14
DT-MD5 (GI)	284444	*aftD*	A	803	0	1	NA	4e-03	+	+	+	+	+	+	7	22	76
	469814	(Rv0390)	D	75	0	1	NA	2e-03	+	402	174	+	132	+	9	+	900
	264910	(Rv0221)	A	282	5	3	0.238	2e-03	+	+	+	+	+	+	10	+	+
	103930	(Rv0094c)	A	245	133	23	0.639	2e-03	+	56	66	44	62	31	11	+	43
	1433251	*oppA*	T	64	1	6	0.117	2e-03	+	+	+	+	+	+	12	+	713
	1482239	(Rv1319c)	W	88	5	1	0.026	4e-04	+	30	52	29	43	73	13	31	112
RF (GI)	607677	(Rv0515)	Y	376	274	218	1.439	2e-03	+	44	24	123	118	+	+	14	932
GBM (GI)	2239885	(Rv1996)	R	294	0	2	0.381	5e-03	+	+	+	+	+	+	+	89	12
	1632134	*PE_PGRS27*	Q	832	3	0	0.044	3e-03	+	+	+	+	+	+	+	389	15
	2480321	*ephD*	S	461	2	0	0.044	2e-03	+	+	190	+	121	+	+	50	18
	1397338	(Rv1251c)	Y	635	1	0	NA	2e-03	+	42	90	54	48	41	+	194	19
	1566623	*dfp*	H	395	1	0	NA	2e-03	+	77	62	+	32	+	+	+	20

S4 Table (continued). ML feature importance of yet unknown variants for EMB

Method	Position	Gene (Locus Tag)	Ref. AA	Codon	p^1g^1	p^0g^1	Dist.	FI value	Rank								
									Lasso	LR-L1	LR-L2	SVM-L1	SVM-L2	DT	DT-MD5	RF	GBM
Lasso (coef.)	866690	*ggtA*	G	234	17	61	0.084	-0.325	6	6	32	10	30	+	+	146	+
	2620781	*dnaG*	S	558	25	15	0.107	-0.268	9	12	26	24	15	+	+	88	311
	174711	(Rv0147)	P	492	12	4	0.051	0.263	10	53	+	8	52	+	+	457	114
	3087532	*ald*	A	238	13	6	0.087	0.209	11	60	+	12	647	+	+	+	141
	1532827	*PPE19*	F	269	2	14	0.469	-0.161	17	+	174	14	681	+	+	283	+
	3447507	*virS*	L	307	58	62	0.053	-0.137	19	10	22	51	42	78	+	18	31
	2644740	*amiA2*	G	141	3	11	0.072	-0.133	20	35	43	37	27	+	+	332	74
LR-L1 (p-val.)	3447507	*virS*	L	307	58	62	0.053	4e-05	19	10	22	51	42	78	+	18	31
	2295685	*pks12*	V	3768	1053	2108	0.982	2e-19	74	3	1	122	174	258	+	136	544
	669033	(Rv0575c)	D	238	1025	2047	0.844	2e-15	+	4	16	+	+	+	+	+	+
	1998063	(Rv1765c)	E	151	743	1438	1.020	3e-09	78	5	10	158	335	7	+	9	14
	2635577	*PPE39*	R	6	237	603	1.071	4e-07	62	7	11	126	51	40	+	8	17
	3883915	(Rv3466)	V	131	92	612	1.006	7e-06	72	8	21	102	124	22	+	35	+
	3811039	(Rv3395c)	N	200	25	15	0.107	8e-04	+	13	25	+	14	201	+	73	707
	2196970	(Rv1945)	H	328	199	173	0.786	1e-03	46	14	19	46	18	+	+	124	+
	908186	*sseC2*	T	100	1016	2069	0.870	2e-03	77	15	8	121	201	+	+	263	933
	371849	*PPE5*	A	306	49	77	0.240	4e-03	31	17	+	20	+	+	+	+	+
	373725	*PPE6*	A	663	49	77	0.240	8e-03	+	18	47	+	+	+	+	+	+
	2196964	(Rv1945)	N	326	189	183	0.829	0.013	92	19	18	151	40	+	+	49	315
	1419658	*lprA*	A	31	169	78	0.125	0.022	57	20	+	148	397	+	+	+	+
LR-L2 (p-val.)	2635577	*PPE39*	R	6	237	603	1.071	1e-05	62	7	11	126	51	40	+	8	17
	2196970	(Rv1945)	H	328	199	173	0.786	6e-03	46	14	19	46	18	+	+	124	+
	4060210	*esxV*	Q	20	1102	2230	1.000	6e-09	+	+	4	144	46	+	+	+	551
	3883796	(Rv3466)	R	91	981	1815	0.759	8e-08	69	+	5	100	31	+	+	314	+
	4060201	*esxV*	S	23	1056	2015	0.996	5e-07	+	+	6	+	304	77	+	94	54
	2264782	(Rv2017)	A	262	1049	2101	0.900	6e-07	101	+	7	67	47	+	+	245	427
	3842620	*PPE57*	T	128	1106	2195	0.997	4e-06	67	+	9	15	344	+	+	290	+
	3135951	(Rv2828c)	S	128	945	1822	0.766	3e-05	134	+	12	191	110	+	+	357	+
	283610	*aftD*	A	1081	1012	2114	0.887	4e-05	+	+	13	131	181	+	+	+	+
	383716	(Rv0315)	P	39	987	2091	0.887	9e-05	+	+	14	+	406	+	+	690	+
	2630740	*plcA*	T	446	1096	2143	0.991	8e-04	+	+	15	163	+	+	+	217	193
	3761191	*PPE56*	V	1971	965	1741	0.714	2e-03	+	+	17	+	140	+	+	446	+
SVM-L1 (coef.)	512239	*ctpH*	G	1028	2	0	0.050	0.475	+	+	317	16	116	+	+	+	+
	3226505	(Rv2917)	D	48	1	0	NA	0.474	+	+	447	17	486	+	+	+	+
	637420	*pitA*	A	15	4	1	0.049	0.465	40	+	128	19	17	+	+	+	159
SVM-L2 (coef.)	2196970	(Rv1945)	H	328	199	173	0.786	-0.117	46	14	19	46	18	+	+	124	+
	2025848	*PPE25*	V	183	7	18	1.248	-0.116	+	+	51	135	20	+	+	227	+
DT (GI)	1657335	*ctpD*	G	125	0	1	NA	0.010	+	+	+	+	+	4	+	39	+
	2029104	*PPE27*	R	227	2	2	1.396	9e-03	+	+	+	+	+	5	5	20	22
	2628253	*plcC*	V	149	21	64	0.089	7e-03	+	+	+	+	+	8	+	41	+
	2192509	(Rv1939)	H	139	1	0	NA	6e-03	+	+	+	+	+	12	+	+	513
	2283528	(Rv2037c)	R	65	40	199	0.959	5e-03	+	+	+	+	+	13	9	25	80
	1789446	(Rv1588c)	V	131	911	1859	0.899	5e-03	90	+	+	174	664	14	10	5	28
	3740016	*PE_PGRS50*	G	920	0	1	NA	5e-03	+	+	+	+	+	16	+	506	+
	2262620	(Rv2015c)	E	151	861	1593	1.002	4e-03	+	+	+	+	+	17	+	13	10
	1278278	(Rv1150)	D	4	856	1552	0.919	4e-03	+	+	+	+	+	18	+	12	21
	2563495	*sseB*	W	108	20	95	0.546	4e-03	83	+	+	128	+	19	+	805	+
	2304351	*pks12*	V	879	0	1	NA	4e-03	+	+	+	+	+	20	+	38	229
DT-MD5 (GI)	1657327	*ctpD*	A	122	1	0	NA	0.017	+	+	+	+	+	+	4	22	+
	3732122	*PPE54*	A	1605	0	13	0.032	6e-03	+	+	+	+	+	28	12	37	596
	2330426	(Rv2073c)	L	180	7	21	0.438	5e-03	47	43	74	77	437	29	13	343	+
	498848	(Rv0412c)	T	258	0	2	6e-03	4e-03	+	+	181	+	159	+	14	+	+
	4193440	*ctpJ*	L	645	0	1	NA	4e-03	+	+	+	+	845	+	15	+	63
	2796952	*PE_PGRS42*	G	145	0	1	NA	4e-03	+	+	+	+	+	+	16	+	+
	4165093	*dnaZX*	A	547	1	3	0.989	3e-03	+	+	+	+	+	+	17	+	+
	2540789	*cyp124*	D	229	0	1	NA	2e-03	+	+	+	+	+	+	18	+	+
	492915	*pta*	D	377	21	64	0.089	2e-03	+	+	+	+	+	+	19	+	+
	1333272	(Rv1190)	T	98	5	4	0.096	2e-03	+	+	+	+	+	+	20	+	+
RF (GI)	401318	(Rv0336)	Y	376	747	1330	1.006	5e-03	+	29	+	209	+	89	+	10	123
	2896527	*aspS*	D	426	1	0	NA	4e-03	+	+	+	+	+	32	+	14	39
	2550014	(Rv2277c)	T	6	129	273	1.158	4e-03	117	+	+	+	+	85	+	15	24
GBM (GI)	26278	(Rv0021c)	A	202	1	1	0.050	0.058	+	+	+	+	478	+	+	+	2
	849500	*PPE12*	G	181	1	1	0.050	0.056	+	+	+	+	480	+	+	+	3
	1561201	*pyrF*	G	253	1	0	NA	0.045	+	+	+	+	476	+	+	+	4
	1061979	*sucC*	Y	6	1	0	NA	0.044	+	+	+	+	479	+	+	+	5
	370764	*PPE5*	L	667	1	3	0.553	0.011	+	+	+	+	+	259	+	162	8
	2075207	(Rv1830)	T	123	3	1	0.317	6e-03	+	+	+	+	+	+	+	+	13
	3132672	(Rv2824c)	R	15	3	1	7e-03	4e-03	+	+	+	+	+	250	+	+	19

S4 Table (continued). ML feature importance of yet unknown variants for PZA

Method	Position	Gene (Locus Tag)	Ref. AA	Codon	p^1g^1	p^0g^1	Dist.	FI value	Rank								
									Lasso	LR-L1	LR-L2	SVM-L1	SVM-L2	DT	DT-MD5	RF	GBM
Lasso (coef.)	4083468	(Rv3645)	M	221	2	0	0.019	0.481	1	46	61	36	20	+	+	51	58
	167711	(Rv0142)	V	1	12	2	0.045	0.437	2	15	45	11	8	61	+	73	36
	4137219	(Rv3695)	V	5	78	31	0.150	0.431	3	5	+	14	11	+	+	152	56
	1731431	*adh*	V	20	4	20	0.133	-0.402	4	19	27	10	15	11	+	7	35
	4097010	(Rv3659c)	W	329	36	7	0.077	0.341	7	25	46	60	7	+	+	6	382
	1254056	(Rv1129c)	D	160	2	6	1.048	-0.304	10	22	35	19	18	33	+	52	46
	2310984	(Rv2052c)	R	512	9	1	0.104	0.303	11	55	205	39	139	+	+	+	901
	3877949	*rpoA*	T	187	10	20	0.660	-0.297	12	9	16	17	4	49	+	36	33
	2180169	(Rv1927)	L	166	8	113	0.053	-0.287	13	7	9	106	10	3	3	2	3
	2520618	*kasB*	A	408	9	0	0.030	0.285	14	63	117	44	82	+	+	+	+
	761314	*rpoB*	F	503	8	1	0.522	0.284	16	71	70	203	45	+	+	+	354
	3087532	*ald*	A	238	15	4	0.087	0.277	17	+	+	66	+	+	+	+	905
	507454	(Rv0421c)	Q	103	72	3	0.113	0.269	18	308	832	521	813	+	+	+	+
	3979119	*PPE63*	A	354	8	0	0.081	0.260	19	51	103	63	52	+	+	+	+
LR-L1 (p-val.)	167711	(Rv0142)	V	1	12	2	0.045	3e-06	2	15	45	11	8	61	+	73	36
	401318	(Rv0336)	Y	376	514	1010	1.006	7e-33	111	3	1	245	95	14	+	25	18
	2517129	*fabD*	T	115	151	95	0.167	3e-23	84	4	17	51	129	+	+	948	+
	3297704	*pks15*	T	46	26	139	0.070	6e-11	76	8	11	65	34	30	+	8	68
	2196970	(Rv1945)	H	328	140	114	0.786	7e-10	86	10	8	214	41	+	+	299	976
	1020942	*PPE14*	S	130	271	612	0.479	4e-09	116	11	10	304	809	+	+	118	797
	3521253	*nuoJ*	V	39	62	42	0.150	4e-09	24	12	+	38	90	+	+	410	428
	1741352	*ansA*	G	281	571	1416	0.742	9e-09	+	13	+	290	+	+	+	124	70
	1235400	*xseA*	G	21	56	16	0.035	1e-06	138	14	39	103	333	+	+	+	+
	3883796	(Rv3466)	R	91	596	1431	0.759	1e-05	161	16	+	+	+	+	+	127	75
	2101193	*ureD*	S	58	67	31	0.147	5e-05	38	18	+	154	+	+	+	658	147
	3883711	(Rv3466)	A	63	8	176	0.970	1e-04	90	20	33	91	46	+	+	15	63
LR-L2 (p-val.)	3877949	*rpoA*	T	187	10	20	0.660	3e-03	12	9	16	17	4	49	+	36	33
	2295685	*pks12*	V	3768	669	1456	0.982	9e-11	+	+	3	+	409	+	+	231	38
	1165114	(Rv1042c)	G	129	653	1391	0.969	2e-08	+	41	4	435	644	31	+	63	302
	4060210	*esxV*	Q	20	688	1508	1.000	1e-07	+	+	5	+	510	+	+	+	+
	908186	*sseC2*	T	100	609	1451	0.870	2e-07	+	+	6	285	351	+	+	787	150
	383716	(Rv0315)	P	39	597	1468	0.887	7e-07	+	+	7	+	+	+	+	943	+
	2196964	(Rv1945)	N	326	133	116	0.829	2e-03	141	+	12	+	549	+	+	314	636
	3447507	*virS*	L	307	8	109	0.053	2e-03	+	+	13	+	22	+	+	13	+
	2635577	*PPE39*	R	6	154	315	1.071	3e-03	131	34	14	343	485	+	+	17	23
	3883915	(Rv3466)	V	131	32	375	1.006	0.016	149	24	20	210	316	71	+	62	312
SVM-L1 (coef.)	2433953	(Rv2172c)	R	195	1	1	0.025	1.808	+	39	199	2	61	35	+	+	30
	189580	*PE4*	T	287	1	0	NA	1.662	+	253	108	3	107	+	+	+	366
	3069203	*hsdM*	H	294	1	0	NA	1.602	+	400	595	4	461	+	+	+	152
	1853636	*tsnR*	T	11	1	0	NA	1.580	+	85	71	5	40	36	+	+	144
	178861	*PE1*	P	150	1	0	NA	1.463	+	43	100	6	42	27	+	148	15
	845017	*mmsA*	G	313	1	0	NA	1.448	+	116	63	7	24	+	+	+	175
	2076475	*gcvB*	T	200	1	0	NA	1.384	+	131	68	9	30	+	+	+	168
	2114591	(Rv1866)	N	484	1	1	0.019	1.290	+	53	87	12	37	77	+	46	43
	2290544	*lipT*	A	226	1	0	0.125	1.213	+	416	723	15	682	+	+	300	+
	1884791	*pks8*	L	1030	2	0	0.087	1.155	74	138	220	16	99	+	+	812	206
SVM-L2 (coef.)	2180169	(Rv1927)	L	166	8	113	0.053	-0.253	13	7	9	106	10	3	3	2	3
	2201720	(Rv1954c)	T	9	36	8	0.146	0.257	+	+	53	+	9	+	+	126	6
	764703	*rpoC*	K	445	10	3	0.292	0.230	35	174	55	142	13	+	+	+	554
	935234	*lpqR*	A	172	4	20	0.133	-0.220	+	26	28	+	16	+	8	32	+
DT (GI)	4370265	*eccD2*	F	474	74	11	0.307	1e-02	+	+	+	+	+	6	+	147	433
	338775	*PE_PGRS4*	A	100	0	2	1.422	1e-02	+	+	254	+	353	7	11	20	31
	1998063	(Rv1765c)	E	151	477	901	1.020	9e-03	179	30	+	425	+	8	+	11	19
	427428	*PPE8*	P	2418	1	44	0.300	7e-03	+	278	+	+	+	12	+	335	+
	3797939	*idsB*	Q	184	0	1	NA	6e-03	+	+	+	+	+	13	+	169	+
	3313568	(Rv2961)	G	96	9	3	0.110	6e-03	+	+	+	+	+	15	+	53	790
	3549270	(Rv3180c)	A	140	0	1	NA	6e-03	+	+	+	+	+	16	+	22	407
	1706685	(Rv1515c)	G	281	597	1436	0.747	6e-03	+	+	+	+	+	17	10	155	+
	3147620	*infB*	M	85	0	2	0.037	6e-03	+	+	+	410	+	18	+	43	795
	432950	*PPE8*	N	577	0	2	0.069	5e-03	+	+	+	+	+	19	+	290	+
	2423286	*PE_PGRS38*	R	518	0	3	0.012	5e-03	+	+	+	+	+	20	+	105	346
DT-MD5 (GI)	935234	*lpqR*	A	172	4	20	0.133	0.012	+	26	28	+	16	+	8	32	+
	720616	*recD*	A	373	2	0	0.019	0.014	+	47	62	21	21	+	6	33	157
	3796887	*lytB1*	Q	184	0	1	NA	0.011	+	+	+	+	+	+	9	156	+
	2119879	(Rv1869c)	P	273	0	4	0.060	7e-03	+	+	+	+	+	+	12	401	+
	3448604	(Rv3083)	L	34	1	0	NA	6e-03	+	117	64	223	25	+	13	81	430
	731943	*hadA*	A	5	1	0	NA	6e-03	+	130	67	258	29	47	14	+	127
	3316380	(Rv2963)	M	382	0	1	0.039	6e-03	+	+	+	+	+	57	15	184	+
	2630740	*plcA*	T	446	680	1465	0.991	6e-03	+	+	+	+	+	+	16	737	+
	1432484	*oppA*	Q	319	2	3	0.107	3e-03	+	+	+	+	+	+	17	+	+
	1582249	*fmt*	I	28	1	41	0.308	3e-03	+	+	+	+	+	+	18	+	+
RF (GI)	2262620	(Rv2015c)	E	151	580	1061	1.002	4e-03	100	+	+	213	54	28	+	12	7
	2915226	*secD*	Y	171	69	4	0.115	3e-03	+	+	+	+	+	+	+	19	39
GBM (GI)	104638	(Rv0094c)	A	9	3	1	9e-03	5e-03	+	123	830	74	154	93	+	385	14
	2986827	(Rv2670c)	A	5	76	23	0.479	5e-03	42	35	+	93	57	+	+	257	17
	2163672	*PPE34*	V	1214	0	1	NA	4e-03	+	+	+	+	+	+	+	34	20

S4 Table (continued). ML feature importance of yet unknown variants for SM

Method	Position	Gene (Locus Tag)	Ref. AA	Codon	p^1g^1	p^0g^1	Dist.	FI value	Rank								
									Lasso	LR-L1	LR-L2	SVM-L1	SVM-L2	DT	DT-MD5	RF	GBM
Lasso (coef.)	1877267	*pks7*	Q	655	19	1	0.126	0.473	3	59	955	+	781	+	+	+	179
	1269567	*echA10*	L	131	62	9	0.111	0.375	4	+	687	5	277	+	+	+	450
	3578696	(Rv3202c)	S	502	3	0	0.071	0.213	5	+	+	+	985	+	+	+	626
	621045	(Rv0530)	T	47	4	2	0.022	0.154	7	+	334	+	192	+	+	79	313
	1169570	(Rv1047)	R	50	57	57	1.006	-0.112	8	15	13	11	29	+	+	61	101
	4370265	*eccD2*	F	474	69	17	0.307	0.083	13	21	+	+	+	+	+	123	514
	4172205	(Rv3726)	L	262	9	0	0.031	0.078	14	+	36	+	5	+	+	+	+
	853066	*phoR*	G	224	4	3	0.024	-0.077	15	+	75	+	53	+	+	100	+
	3712581	(Rv3327)	S	278	10	17	1.491	0.072	18	+	200	+	587	+	+	12	+
	1030058	(Rv0923c)	T	174	22	7	0.463	0.070	19	+	457	+	743	+	+	524	+
	2025913	*PPE25*	S	205	51	14	0.687	0.061	20	+	59	+	62	+	+	210	216
LR-L1 (p-val.)	1169570	(Rv1047)	R	50	57	57	1.006	0.186	8	15	13	11	29	+	+	61	101
	908186	*sseC2*	T	100	556	774	0.870	1e-05	43	4	+	12	+	+	+	97	+
	3883915	(Rv3466)	V	131	48	292	1.006	3e-03	30	5	21	19	28	5	5	4	5
	1739559	*ileS*	I	1014	244	35	0.438	1e-02	39	6	733	10	696	+	+	95	+
	773497	*echA4*	F	125	566	737	0.845	0.022	65	10	+	16	+	+	+	+	+
	141623	*oxyS*	D	142	566	737	0.844	0.022	62	10	+	17	+	+	+	+	+
	3664457	(Rv3282)	A	80	566	737	0.844	0.022	21	10	+	36	+	+	+	+	+
	1341114	*esxL*	T	37	11	18	0.977	0.175	22	14	52	23	586	+	+	69	+
	607677	(Rv0515)	Y	376	102	170	1.439	0.244	33	16	4	24	10	+	+	8	301
	401318	(Rv0336)	Y	376	420	441	1.006	0.256	45	17	6	31	34	+	+	35	343
	1533381	*PPE19*	E	85	11	33	0.637	0.264	24	18	98	22	524	7	+	22	+
	2053560	*mgtC*	A	40	18	39	0.590	0.303	28	19	+	21	+	+	+	44	+
	669033	(Rv0575c)	D	238	565	735	0.844	0.370	+	20	+	+	+	+	+	682	+
LR-L2 (p-val.)	607677	(Rv0515)	Y	376	102	170	1.439	1e-05	33	16	4	24	10	+	+	8	301
	2262620	(Rv2015c)	E	151	544	569	1.002	7e-06	40	60	3	33	12	+	+	101	464
	1165114	(Rv1042c)	G	129	594	693	0.969	2e-04	47	+	7	+	30	30	+	18	15
	1998063	(Rv1765c)	E	151	479	532	1.020	0.011	55	+	9	+	76	+	+	24	395
	1341174	*esxL*	L	57	2	0	0.025	0.050	23	+	10	+	6	8	8	392	20
	766488	*rpoC*	P	1040	6	0	0.766	0.058	31	+	11	+	3	+	+	+	25
	178861	*PE1*	P	150	1	0	NA	0.177	+	+	15	+	8	17	+	+	23
	3135951	(Rv2828c)	S	128	503	568	0.766	0.200	+	+	16	+	48	+	+	359	213
	3111531	(Rv2802c)	F	98	0	1	NA	0.207	+	+	17	+	13	+	+	+	45
	2288678	(Rv2042c)	A	2	1	0	NA	0.209	+	+	18	+	7	19	+	+	69
	2550014	(Rv2277c)	T	6	88	128	1.158	0.251	50	+	19	+	92	+	+	109	778
	2959130	(Rv2632c)	D	21	2	1	0.012	0.254	+	+	20	+	21	49	+	266	49
SVM-L1 (coef.)	1332899	*sigI*	K	270	20	1	0.124	0.642	+	27	938	3	760	+	+	+	247
	2151222	(Rv1904)	L	90	579	795	0.881	-0.145	+	+	+	8	+	+	+	+	+
	2300546	*pks12*	H	2147	650	829	1.000	-0.115	+	+	+	13	+	+	+	+	+
	2296042	*pks12*	P	3649	658	827	0.999	-0.115	+	+	+	14	+	+	+	+	90
	103756	(Rv0094c)	P	303	531	725	0.889	-0.101	64	+	51	15	425	+	+	59	55
	2295685	*pks12*	V	3768	626	758	0.982	-0.073	44	+	25	20	424	+	+	+	630
SVM-L2 (coef.)	1341174	*esxL*	L	57	2	0	0.025	0.368	23	+	10	+	6	8	8	392	20
	766488	*rpoC*	P	1040	6	0	0.766	0.484	31	+	11	+	3	+	+	+	25
	178861	*PE1*	P	150	1	0	NA	0.292	+	+	15	+	8	17	+	+	23
	2288678	(Rv2042c)	A	2	1	0	NA	0.321	+	+	18	+	7	19	+	+	69
	852682	*phoR*	L	96	0	1	NA	-0.221	+	+	39	+	15	+	+	+	129
	4138789	*glpK*	I	323	0	2	6e-03	-0.217	+	+	24	+	16	+	+	+	46
	990270	(Rv0890c)	L	776	0	1	NA	-0.205	+	+	40	+	18	14	+	+	8
	110112	*nrp*	E	38	1	0	NA	0.201	+	+	22	+	19	+	+	+	+
	4033055	*PE_PGRS58*	P	35	1	0	0.025	0.200	+	+	29	+	20	46	+	+	106
DT (GI)	1341174	*esxL*	L	57	2	0	0.025	8e-03	23	+	10	+	6	8	8	392	20
	4407982	*gid*	L	74	0	1	NA	0.019	+	+	+	+	+	4	+	9	788
	3890330	*bpoA*	N	135	5	1	0.238	0.014	+	+	+	+	+	6	6	7	167
	3844586	(Rv3427c)	G	19	1	0	NA	8e-03	+	+	+	+	+	9	+	73	+
	2338332	(Rv2081c)	S	58	1	3	0.581	7e-03	+	+	+	+	+	10	+	+	14
	3929794	*PE_PGRS53*	N	1076	0	1	NA	6e-03	+	+	+	+	+	11	+	278	24
	3950177	*PE_PGRS57*	T	1462	0	6	0.116	6e-03	+	+	+	+	+	12	+	99	17
	1972331	*pknF*	A	65	1	1	0.019	6e-03	+	+	+	+	+	13	11	+	13
	2044509	*PPE30*	A	376	1	15	0.436	5e-03	+	+	+	+	+	15	+	+	197
	274904	(Rv0229c)	H	28	1	0	NA	4e-03	+	+	44	+	27	18	+	+	44
	3823954	(Rv3404c)	W	211	1	0	0.243	4e-03	+	+	402	+	409	20	+	+	+
DT-MD5 (GI)	4408120	*gid*	G	28	1	0	NA	0.021	+	+	+	+	+	+	4	10	309
	1532855	*PPE19*	S	260	0	9	0.475	0.014	+	+	+	+	+	+	7	58	+
	589583	(Rv0499)	A	134	0	1	NA	8e-03	+	+	689	+	699	+	9	+	414
	3929842	*PE_PGRS53*	A	1092	1	0	NA	7e-03	+	+	+	+	+	+	10	+	425
	3826382	*vapB47*	R	44	3	1	0.115	4e-03	+	+	+	+	+	+	12	+	+
	2299269	*pks12*	G	2573	0	2	0.044	4e-03	+	+	+	+	+	+	13	+	+
	1097758	*mprB*	A	84	0	1	NA	4e-03	+	+	+	+	+	+	14	+	+
	4377290	*eccC2*	R	1055	0	2	0.094	3e-03	+	+	+	+	+	+	15	330	+
	4312621	*pheA*	A	17	4	35	0.104	2e-03	+	+	+	+	+	+	16	+	+
	437119	(Rv0358)	P	87	0	3	0.042	2e-03	+	+	+	+	+	+	17	+	842
	1659466	*trxB1*	C	33	0	1	NA	2e-03	+	+	+	+	+	+	18	+	840
RF (GI)	4408107	*gid*	E	32	2	0	0.432	6e-03	+	+	932	+	916	+	+	5	4
	4407995	*gid*	S	70	1	0	1.760	4e-03	+	+	+	+	+	+	+	6	+
	2635577	*PPE39*	R	6	160	239	1.071	3e-03	51	+	27	35	42	+	+	11	139
	4220356	*lipE*	W	224	1	4	0.571	2e-03	+	+	+	+	+	+	+	13	+
	2950652	(Rv2624c)	T	219	0	3	0.043	2e-03	+	+	+	+	+	+	+	14	+
	2040648	*PPE28*	Y	399	0	1	NA	2e-03	+	+	+	+	+	+	+	15	+
	1278278	(Rv1150)	D	4	459	443	0.919	2e-03	+	+	56	+	147	38	+	17	54
	2915226	*secD*	Y	171	59	10	0.115	2e-03	+	+	57	+	631	+	+	19	18
	4233356	(Rv3786c)	A	81	1	0	NA	2e-03	+	+	+	+	+	+	+	20	348
GBM (GI)	1532859	*PPE19*	A	259	0	9	0.475	7e-03	+	+	+	+	+	+	+	43	7
	3844372	(Rv3427c)	R	90	0	1	NA	6e-03	+	+	+	+	+	+	+	64	9
	155565	*mak*	P	445	4	2	0.022	6e-03	+	+	339	+	196	+	+	122	10
	4134023	*moxR2*	R	170	0	1	NA	6e-03	+	+	+	+	+	+	+	+	12
	4274541	*pirG*	P	268	240	32	0.438	5e-03	+	62	+	+	+	+	+	85	16
	2040753	*PPE28*	T	434	1	0	NA	4e-03	+	+	+	+	+	+	+	+	19
