# Supplementary material for: Robust detection of point mutations involved in multidrug-resistant Mycobacterium tuberculosis in the presence of co-occurrent resistance markers
Source: PLoS Comput Biol. 2020 Dec 21;16(12):e1008518. doi: 10.1371/journal.pcbi.1008518 (PMC7785249; doi:10.1371/journal.pcbi.1008518)
Supplement: S3 Methods — (PDF) [file pcbi.1008518.s016.pdf]

Supplementary Methods

HHS algorithm

Algorithm 1: Cannibalistic elimination

```
set effect size as defined by user
calculate the sum of all positive scores
for iteration do
    SNPs ← SNPs[scores > 0]
    for SNP_i in SNPs do
        p^1g^1_i strains ← strains[p = 1 and g_i = 1]
        strength_i ← number of p^1g^1_i strains · score_i
        for SNP_j in SNPs do
            get number of strains in p^1g^1_i strains with p = 1 and g_j = 1
            strength_j ← number of strains · score_j
            if j ≠ i then
                strength_i ← strength_i − strength_j · effect size
            end
        end
        new score_i ← strength_i / number of p^1g^1_i strains
    end
    rescale all scores so that their sum matches the sum calculated initially
end
```

Initial score example

To illustrate how scores are calculated in the initial phase, we will go through the process for the SNP at position 2155168 (*katG* S315) for resistance against INH with 3501 samples (1942 resistant; 1559 susceptible). In addition to determining $p^1g^1 = 1676$ (the number of resistant samples with the SNP) and $p^0g^1 = 12$ (the number of susceptible samples with the SNP), the program also computes the relative average pairwise distance among all resistant samples that feature the SNP ($d_{p^1g^1} = 0.94456$ in this case).

In the next step, p^1g^1 and p^0g^1 are normalised: The factors for normalisation are calculated according to $\frac{\text{number of samples}}{\text{number of resistant (or susceptible) samples}\cdot 2}$ so that the effect of an imbalanced phenotype / genotype is removed. Thus, the weights for the resistant and susceptible phenotype are $w_{p^1} = \frac{3501}{2\cdot 1942} = 0.90139$ and $w_{p^0} = \frac{3501}{2\cdot 1559} = 1.1228$, respectively. Similarly, the genotype weights are

$$w_{g^1} = \frac{3501}{2\cdot 1688} = 1.0370, \qquad w_{g^0} = \frac{3501}{2\cdot 1806} = 0.96927.$$

Note, that in the genotype case the sum of the relevant samples does not equate to the total number of samples ($1688 + 1806 = 3494 < 3501$) due to a few missing genotypes.

Now, the score for the normalised counts can be determined:

$$\begin{aligned} \text{score} &= (p^1g^1 \cdot w_{p^1} \cdot w_{g^1} - p^0g^1 \cdot w_{p^0} \cdot w_{g^1} \cdot w_{p^0g^1}) \cdot d_{p^1g^1} \\ &= (1676 \cdot 0.90139 \cdot 1.0370 - 12 \cdot 1.1228 \cdot 1.0370 \cdot 2) \cdot 0.94456 \\ &= 1538.7 \cdot 0.94456 \\ &= 1453.4 \end{aligned}$$

with an extra weight $w_{p^0g^1}$ to penalise samples that are susceptible despite carrying the SNP.

Parameter selection

In addition to the number of iterations and the 'delta' value (the relative magnitude of changes per iteration), the user can set multiple additional parameters that affect how the initial scores are determined. The full parameter set includes:

- The number of iterations and
- the iteration effect size (or step size) 'delta'. These two are tied together as smaller deltas need more iterations to converge. The default value for delta (1.0×10^{-3}) proved sufficiently small in most situations. However, sometimes more than 100,000 iterations might be required for convergence. This is not a big issue, though, as computational complexity scales with the square of the number of active SNPs and these tend to be relatively few in the later stages of elimination (or after convergence). Hence, since most of the heavy lifting is done right after the start it is cheap to select a higher number of iterations. Future versions of HHS will stop as soon as there is only minimal change in the scores or allow the user to resume a previous run. However, we have not implemented these features at this time.
- The way how genotype weights are calculated: either for the full dataset as a whole or on a per-SNP basis. We recommend the SNP-wise option, which is also the default in the command line program.
- The relative strength of the genotype weights (i.e. a continuous setting from 0.0 to 1.0 for how strongly the imbalance in the genotype should be taken into account). In order to give rare variants a chance we recommend values above 0.5.
- The relative strength of the phenotype weights. We have not experimented much with this setting and suppose it does not affect the results much as long as the phenotype vector is somewhat reasonably balanced. Thus, we would recommend the default of 1.0.
- The penalty factor for susceptible strains with the genotype ($w_{p^0g^1}$). This is arguably the most important setting as it controls how rigorously SNPs that occur in many susceptible samples should be weeded out. One could argue that such SNPs constitute false positives and thus would be removed in the elimination phase anyway. However, we had best results when setting this value to 2.0.
- The relative strength of the average pairwise distance $d_{p^1g^1}$. Like the other weight factors, this can be dialled continuously from 0 (completely ignore the distance value; $d_{p^1g^1} = 1$) to 1 ($d_{p^1g^1}$ represents the actual distance). As a surrogate for incorporating population stratification the average pairwise distance is crucial in order to obtain valid initial scores. We therefore recommend leaving this value at its default, 1.0.
- A filter for p^1g^1 (initial scores of SNPs that appear in fewer resistant samples are set to 0). SNPs that occur very rarely (e.g. only twice in the whole dataset) tend to have very large initial scores when the relative strength of the genotype normalisation weights is close to 1.0. This is especially the case when the two samples featuring the SNP reside on opposite sides of the phylogenetic tree ($\rightarrow$ close to maximum average pairwise distance). In addition to very large initial scores, these rare variants have another competitive advantage since they overlap with fewer other SNPs as opposed to their more common counterparts. Thus, in order to prevent rare variants from dominating the elimination phase when the genotype weight is set to 1.0, we recommend setting this value to 3 or higher (i.e. remove all SNPs that occur in fewer then three resistant samples). The actual value should be chosen with respect to the size of the dataset. We had good results with 5 and datasets with a few thousand samples.

In the study presented here, for every single-drug dataset the following combinations of parameters were run once with P0G1_extra_weight=2 and once with P0G1_extra_weight=10.

- default
- P1G1_filter=5
- dist_weight=0

- rel_gt_weight=0
- P1G1_filter=5, dist_weight=0
- P1G1_filter=5, rel_gt_weight=0
- P1G1_filter=5, rel_gt_weight=0, dist_weight=0

All results can be found in S4 Data.
